# Training strategy over architecture: A systematic ablation study for *Spartina* detection from aerial imagery with small geospatial datasets

**DOI:** 10.1371/journal.pone.0358464

**Published:** 2026-09-18

**Authors:** Adrien Le Guillou, Manon Brehier, Jérôme Ammann, Xavier Dauvergne, Pierre Stéphan

**Affiliations:** 1 Univ Brest, Univ Nantes, Univ Rennes, CNRS, LETG UMR, IUEM, Plouzané, France; 2 Geo-Ocean, UMR CNRS-IFREMER-UBO-UBS, IUEM, University of Brest, Plouzané, France; 3 Laboratoire Géoarchitecture: Territoires, Urbanisation, Biodiversité, Environnement, Université de Brest, Brest, France; The University of Auckland - City Campus: University of Auckland, NEW ZEALAND

## Abstract

In operational coastal monitoring of spatially fragmented intertidal habitats, labelled datasets are structurally constrained by the limited spatial extent of target species, yet the relative influence of training strategy versus architecture on segmentation performance remains poorly characterised. We present a systematic ablation study disentangling these contributions for automated detection of the invasive cordgrass *Spartina* from very high resolution aerial imagery. Three segmentation architectures, U-Net, DeepLabV3 + , and SegFormer-B2, were trained under twelve configurations spanning four training-strategy axes, each evaluated by five-fold cross-validation on 810 patches derived from aerial colour-infrared imagery and elevation data, yielding 180 training runs. Training strategy dominates architectural design: the gap between the best and worst strategy (~10 pts of overlap accuracy) exceeds the inter-architecture spread (<1 pt) by an order of magnitude, and encoder freezing and the Tversky loss were systematically detrimental across all three architectures. The selected operational configuration achieves an F1-score of 86.8±1.3%, enabling wall-to-wall surface estimation across the Bay of Brest (44.3 ha; uncertainty ±10–15%). A channel ablation shows spectral information dominates: removing elevation costs only 1.7 pts, whereas elevation alone still reaches 54.2±3.1%; this likely reflects limitations of fusing a heterogeneous modality into a photographically pre-trained encoder rather than the ecological irrelevance of elevation. A leave-one-site-out validation, withholding each labelled site in turn, yields a lower and more variable estimate (F1 77.8±6.9%, ranging 50.5–79.5% across sites), indicating the five-fold estimate is optimistic for genuinely new sites. On an independent site never used in training, predictions agree with manual photo-interpretation of the same imagery to within 2.4%, though independent field validation is still needed. Derived from routinely updated national imagery, this pipeline offers coastal managers a reproducible, annually updatable monitoring tool, best suited to sites resembling those already mapped.

## 1. Introduction

*Spartina* (now named *Sporobolus*) is an invasive macrophyte that has been progressively colonising the estuaries, bays, and intertidal flats of the French Atlantic coast since the 1950s–1960s [1,2]. The ecological status of this species is paradoxical from an ecosystem services perspective. On one hand, *Spartina* is recognised as an efficient blue carbon sink, sequestering organic carbon at rates comparable to or exceeding those of other coastal wetland ecosystems such as wet meadows [3,4], and its dense root systems contribute to reducing coastal flooding risk by stabilising low-marsh mudflats and play a documented role in the long-term geomorphic evolution of estuarine wetlands [5]. On the other hand, at the scale of French intertidal ecosystems, *Spartina* is primarily regarded as an ecological threat: its aggressive colonisation of the low schorre competes directly with native halophytic species including *Limonium* spp., *Halimione portulacoides*, and reed communities and degrades the habitat quality for specialised intertidal flora and fauna, as well as for waterbird populations dependent on open mudflat foraging areas [6,7].

Accurate and repeated spatial monitoring of *Spartina* is therefore a prerequisite for effective management of French coastal wetlands: recent evidence based on dense time-series satellite imagery demonstrates that tidal flat structural stability responds rapidly to large-scale eradication campaigns, underscoring the need for temporally consistent and automated mapping frameworks [8]. However, field-based cartography is inherently challenging: the species can be visually ambiguous even at close range, particularly in mixed assemblages where it co-occurs with other halophytic species such as *Halimione portulacoides*, *Limonium*, and hybrid transitional communities. Furthermore, the intertidal surfaces affected in semi-enclosed embayments such as the Bay of Brest (BoB) are spatially limited compared to large *Spartina*-dominated systems documented in China or the United States [9–11], which restricts the volume of labelled training data that can realistically be collected. These constraints, small spatial footprint, spectral class ambiguity, and structurally limited dataset size, are not incidental to this study but constitute its primary experimental condition: *Spartina* detection in the Bay of Brest is deliberately used here as a controlled benchmark for evaluating deep learning behaviour under the data scarcity regime that coastal managers actually face, rather than under the large-scale, multi-temporal dataset conditions available in other geographic contexts.

Semantic image segmentation has emerged as a robust methodological framework for the automatic detection and spatialisation of vegetation from aerial and satellite imagery [12–15]. Recent advances in deep learning have produced three structurally distinct families of segmentation architectures: classical symmetric encoder–decoder convolutional networks (U-Net; [16]), dilated convolutional networks with multi-scale pooling (DeepLabV3 + ; [17]), and attention-based hierarchical Transformer architectures (SegFormer; [18]). While comparative benchmarks of these architectures exist in the remote sensing literature, the influence of training strategy on model behaviour and how this influence varies across architectural families remains poorly understood in the context of small, imbalanced, geographically complex datasets. Foundation models (e.g., SAM [19], Prithvi [20]) represent a promising direction but do not yet provide sufficiently targeted responses for fine-grained detection of geographically constrained objects at the operational scales relevant to coastal managers.

While several studies have benchmarked segmentation architectures for remote sensing applications [21–23], these comparisons typically evaluate models under a single, fixed training protocol, implicitly assuming that architectural ranking is invariant to hyperparameter choices. This assumption has been questioned in adjacent domains notably medical image segmentation, where Ma et al. [24] demonstrated that loss function selection alone can shift performance by 5–7% Dice across architectures but has not been systematically tested in the context of small, domain-shifted geospatial datasets.

The present study addresses this gap by providing, to our knowledge, the first controlled ablation framework that isolates the effect of training strategy from architectural design across three structurally distinct segmentation families, under the realistic data constraints of operational coastal vegetation mapping. Three representative architectures (U-Net, DeepLabV3 + , SegFormer-B2) were trained under twelve distinct configurations varying four training strategy axes: data augmentation policy, loss function, regularisation scheme, and optimisation strategy. Each configuration was evaluated by five-fold cross-validation on a dataset of 810 image patches derived from IGN BD ORTHO^®^ colour-infrared (CIR) orthoimagery and a high-resolution digital surface model (DSM) at 20 cm ground sampling distance (GSD), yielding 180 training runs in total. The specific objectives are:

(i) to identify which training strategies consistently improve or degrade segmentation performance across all three architectural families;
(ii) to characterise architecture-specific sensitivities to individual training parameters, with a focus on the interaction between regularisation and internal architectural mechanisms;
(iii) to provide actionable guidance, explicitly bounded to the small-dataset, domain-shift conditions of fragmented intertidal systems, for practitioners developing automated *Spartina* mapping pipelines from routinely updated aerial imagery, with direct applicability to coastal managers such as the Parc Naturel Régional d’Armorique (PNRA).

The overarching aim is not to produce a universally generalisable model nor to benchmark architectures on a large-scale dataset, but to characterise the behaviour of distinct deep learning paradigms under the realistic constraints of limited, spatially fragmented, and ecologically heterogeneous training data. The conclusions are explicitly bounded to this regime: the recommendations derived here are intended for practitioners operating under similar data scarcity conditions in fragmented coastal systems, and their transferability to other estuarine contexts remains to be validated through future multi-site campaigns.

## 2. Materials and methods

### 2.1. Study area

**Bay of Brest: a semi-enclosed macrotidal embayment.** The Bay of Brest (BoB) is a semi-enclosed macrotidal embayment that covers approximately 180 km^2^ at the western tip of the Armorican Massif. Its morphology is characterised by a highly indented coastline inherited from the Holocene marine transgression, which partially inundated an Appalachian-type relief of alternating ridges and valleys, producing a succession of drowned estuaries separated by rocky headlands. This irregular configuration results in a mosaic of sheltered intertidal environments such as salt marshes, mudflats, and macrophyte-dominated low-marsh zones that are spatially fragmented and individually small compared to other European Atlantic systems. The tidal range reaches up to 8 m during spring tides, generating strong current gradients that influence sediment dynamics and vegetation zonation across the intertidal gradient.

The intertidal areas of the BoB are designated as Natura 2000 habitats of community interest, notably due to the presence of the endemic *Limonium humile* (lax-flowered sea lavender), a protected halophyte restricted to the mid-schorre of several Brittany estuaries. The conservation of these communities is directly threatened by the progressive colonisation of the intertidal flats by *Sporobolus alterniflorus* (syn. *Spartina alterniflora*), which currently occupies up to 85% of saltmarsh surfaces in the BoB [25,26] and is present in 100% of *Limonium humile* sites, growing in direct contact with the latter in 94% of monitored stations [27].

**Fig 1:**
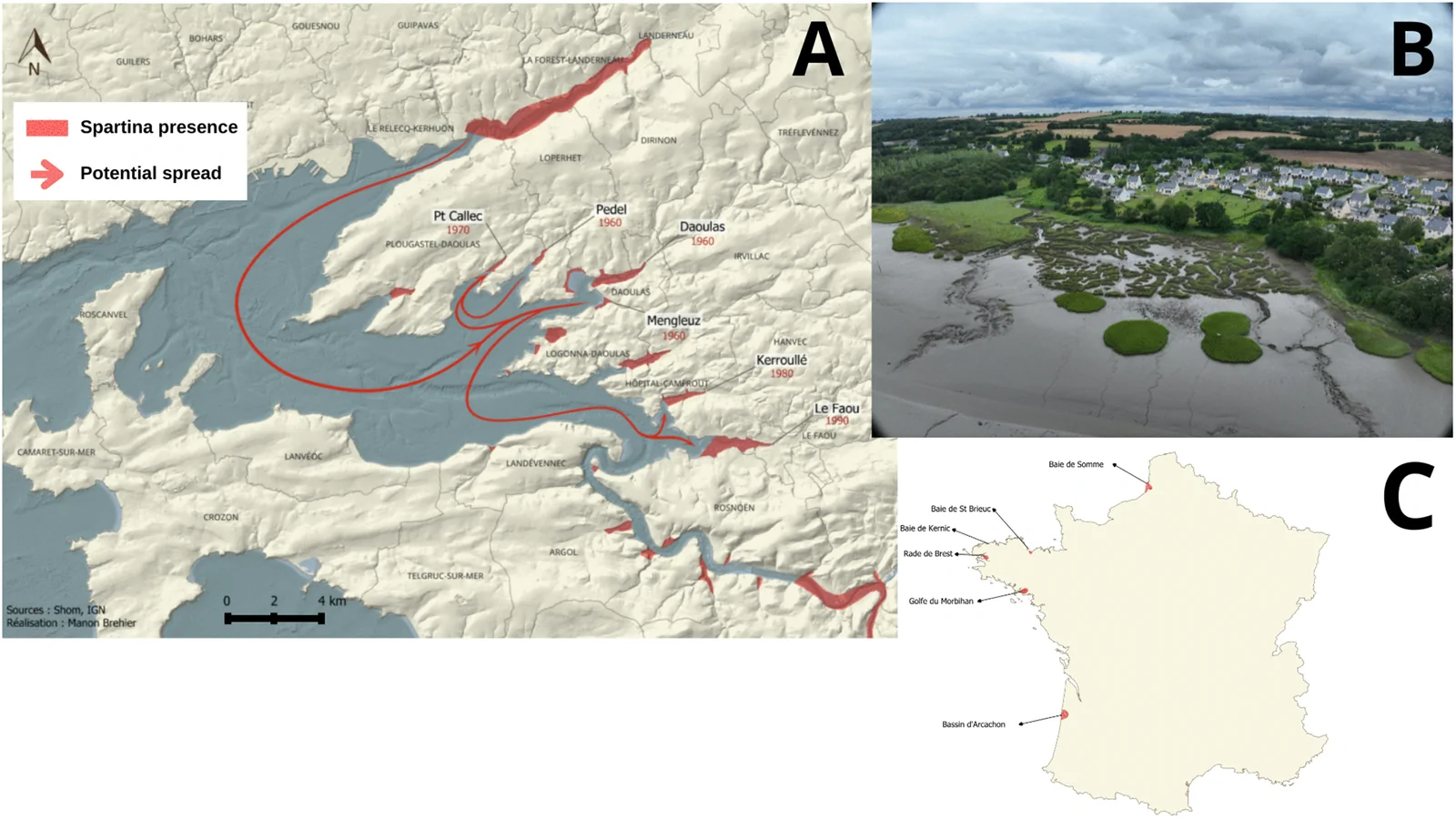
Spatial distribution and morphological characterisation of *Spartina* spp. (A) Map of study sites in the Bay of Brest showing confirmed occurrences and assumed expansion areas. (B) Drone-based aerial view of *Spartina* rhizome structures in a transitional estuarine mudflat (Bay of Brest). (C) Regional distribution of *Spartina* along the French Atlantic and Channel coastlines. Basemap imagery in panel (A): SHOM, IGN, Licence Ouverte/Etalab 2.0. Panel (B) image source: Pierre Stéphan (2024).

***Spartina* invasion history in the Bay of Brest.** *Spartina* was first described in the BoB along the Elorn estuarine shoreline, and its expansion has since been monitored by the Conservatoire Botanique National de Brest (CBNB), which documented a marked acceleration from 1970 onwards [27]. The species subsequently spread across the BoB through a series of successive dispersal events, colonising the Daoulas and the Pédel estuary around 1960, Pont-Callec around 1970, Kerroullé around 1980, and the Faou estuaries around 1990 [28]. This stepwise southward progression is consistent with the fragmented morphology of the BoB: the alternation of deep estuarine inlets and exposed rocky headlands likely slowed dispersal by limiting the connectivity between intertidal mudflat habitats (Fig 1).

Long-term diachronic mapping has provided quantitative evidence of this expansion. A first cartographic study, covering the 1952–2000 period through digital photo-interpretation of historical IGN aerial photographs combined with differential GPS (DGPS) ground surveys, was conducted by Sparfel et al. [29] for three reference estuaries (Pont-Callec, Pedel, and the Mengleuz/Rosmélec salt-marshes, the latter forming part of the broader Daoulas estuarine system). Subsequent surveys were carried out in 2014, covering the entire inner Bay [30], and in 2024, when the diachronic dataset of Sparfel et al. [29] was updated for the Pont-Callec and Pedel sectors and extended to the Faou estuary [31]. By the mid-2000s, vegetation inventories conducted in the context of the Natura 2000 network estimated that *Spartina* dominated communities accounted for 60–75% of slikke and schorre vegetation surfaces in the Bay and along the Elorn river [32,33], rising to 85% according to the 2014 management plan [25].

Spartina in BoB serves as a compelling geographical case study, raising critical questions that are often overlooked in applied remote sensing. While classified as an invasive species, *Spartina* also contributes to ‘Blue Carbon’ sequestration and provides expanded ecological niches for certain mudflat species [4]. This ecological dichotomy warrants careful discussion, as the spatio-temporal dynamics of coastal wetlands can be more complex to interpret than they initially appear. Le Guillou and Niculescu [34] identified an increasing trend in salt marsh surface area across Brittany; however, this expansion is not necessarily synonymous with ecosystem health. The spread of *Spartina* exemplifies this paradox: a net gain in vegetation cover does not always translate into a positive ecological outcome [3,10].

Despite the implementation of experimental eradication measures on priority sites selected on the basis of ecological stakes related to *Limonium humile* conservation no systematic assessment of their long-term effectiveness has yet been conducted. In response, the Parc Naturel Régional d’Armorique (PNRA) and associated research partners have initiated a new management framework integrating Nature-based Solutions (NbS), with the dual objective of pursuing diachronic monitoring and adapting intervention strategies to local ecological priorities.

**Fig 2:**
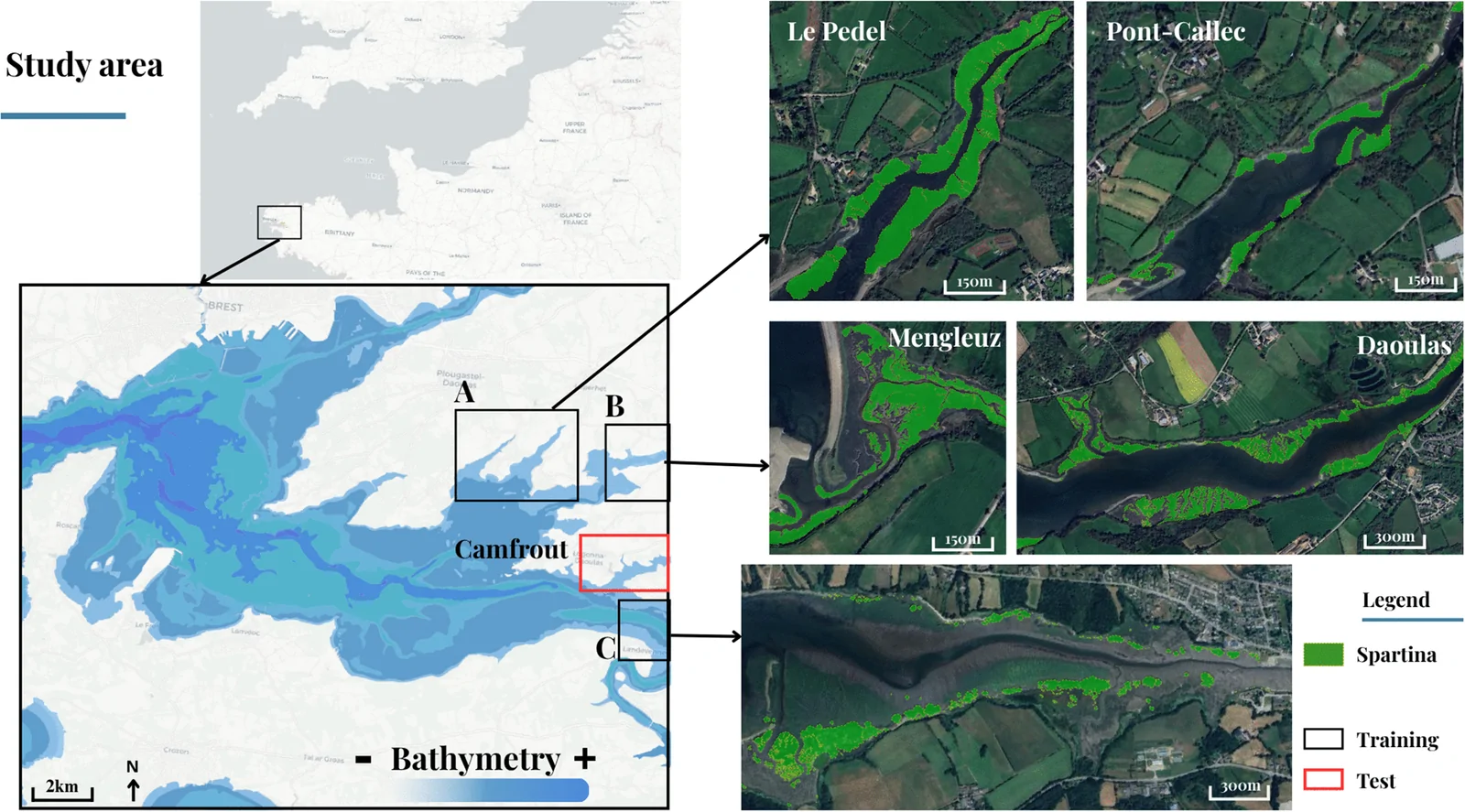
Location of the study area and spatial distribution of the dataset sites. Location of the study area in the Bay of Brest (Brittany, France) and spatial distribution of the *Spartina* dataset sites. Black areas represent the training/validation sets, and the red area indicates the independent test site used for final model evaluation. Basemap: IGN BD ORTHO^®^, Licence Ouverte / Etalab 2.0.

**Study sites.** The present study draws on six labelled zones distributed across the BoB (Fig 2), each corresponding to an estuary or tidal inlet of varying extent: the **Daoulas estuary**, **Mengleuz/Rosmélec**, **Le Faou**, **Keroullé**, **Le Pédel**, and **Pont-Callec**. A point of nomenclature should be clarified at the outset, as it is a recurring source of ambiguity in the local literature: Mengleuz/Rosmélec is a distinct inlet, but it lies within the broader Daoulas estuarine system and is therefore sometimes reported as part of Daoulas rather than as a separate entity. Throughout this article the six zones listed above are treated as distinct labelled units, except where explicitly stated otherwise (in particular, the leave-one-site-out analysis of Section 3.5 groups Daoulas and Mengleuz/Rosmélec into a single spatial unit, following the site boundary polygons used for patch extraction).

The **Daoulas estuary** is a sheltered macrotidal estuary located on the south-eastern shore of the Bay. It constitutes one of the most emblematic *Spartina* invasion fronts in the Brest area, with colonisation documented from the early 1960s. The estuary hosts significant populations of *Limonium humile* on its mid-schorre terraces, making it a priority site for conservation management under the Natura 2000 framework.

The **Mengleuz/Rosmélec estuary** is a smaller semi-enclosed tidal estuary on the eastern shore of the BoB, originally studied by Sparfel et al. [29]. It provides a complementary morphological context characterised by a narrower tidal prism and a more confined intertidal area, allowing assessment of model generalisation across different estuarine configurations.

The **Le Faou estuary** is a macrotidal estuary on the south-eastern shore of the Bay, comparable in scale to Daoulas and contributing the second largest share of labelled patches. **Keroullé**, **Le Pédel**, and **Pont-Callec** are smaller inlets and estuarine embayments; the latter two were among the reference sites of the historical diachronic survey of Sparfel et al. [29], and provide morphological contexts with limited intertidal extent. Together, these six zones span a gradient of estuarine size and confinement, from extensive macrotidal systems to narrow sheltered inlets.

Together, these sites are representative of the spatially constrained intertidal morphologies that characterise *Spartina* habitats in the Bay and, more broadly, in many semi-enclosed Atlantic coastal systems of France. Their limited spatial extent relative to large *Spartina* dominated systems in China or the United States and the complex spectral mixing between the target species and adjacent halophytic communities make them a demanding but ecologically relevant benchmark for automated deep learning-based mapping at very high spatial resolution.

### 2.2. Ethics statement and permits

This study did not involve human subjects, and no informed consent procedure was therefore required. Fieldwork consisted exclusively of non-invasive vegetation surveys (GNSS/DGPS mapping of plant community boundaries and *Limonium humile* occurrences) conducted on foot on the intertidal foreshore (estran) of the Bay. The land accessed is public, not privately owned: it forms part of the French domaine public maritime, and under French law pedestrian access for this type of non-invasive ecological survey is free and does not require specific authorisation (Article L.321−9 of the Code de l’environnement; Article L.2124−4 of the Code général de la propriété des personnes publiques). Part of the study area is additionally protected as the Natura 2000 site “Rade de Brest, estuaire de l’Aulne” (ZSC FR5300046); the surveys conducted did not involve sampling or removal of protected species and did not correspond to activities on the local reference list requiring a formal Natura 2000 impact assessment (Article L.414−4 and Article R.414−19 *et seq.* of the Code de l’environnement). Drone flights were conducted in accordance with French civil aviation regulations, with the corresponding flight authorisations obtained in advance from the relevant military and civil aviation authorities. No approval from an institutional review board or animal ethics committee was required, as this study involved neither human participants nor regulated animals.

### 2.3. Dataset

**Ground truth labelling.** Producing accurate ground truth labels for *Spartina* in the BoB context is inherently challenging. The species forms narrow, discontinuous colonisation fronts at the low marsh margin, often interdigitating with spectrally similar halophytes (*Halimione portulacoides*, *Limonium* spp.) and bare mudflat surfaces. Boundary delineation therefore requires either direct field observation or very high resolution imagery that resolves individual plant tussocks with a level of detail that routine aerial survey products cannot provide alone. To address this constraint, ground truth data were assembled from three complementary sources, each covering a distinct temporal window.

A multi-stage field campaign was conducted in 2025 to produce a high-accuracy cartographic reference for the present study area. In March 2025, vegetation fronts were mapped along the shores of the Daoulas estuary and Lanveur bay using two GNSS receivers (Trimble GPS and SparkFun Facet DGPS in RTK mode), referenced to the IGN69 altimetric system via the Centipède network and reprojected to Lambert-93 (EPSG:2154). In April 2025, two photogrammetric drone flights were conducted over the Daoulas estuary (senseFly eBee X downstream; DJI Mavic 3E upstream) and an additional flight over the Mengleuz/Rosmélec estuary, producing centimetric-resolution orthophotographs and digital elevation models processed in Agisoft Metashape with 18 GNSS-surveyed ground control points per flight. In June 2025, complementary surveys were carried out with the CBNB to map *Limonium humile* stations and refine priority management zones.

These drone orthophotographs and field measurements served exclusively as a labelling reference: they were used to produce and verify polygon delineations of *Spartina* at high spatial accuracy, but were *not* used as model inputs (see Section 2.3). It should also be noted that drone coverage was not available for all labelled sectors of the study area: several intertidal zones, particularly those mapped through diachronic photo-interpretation were digitized solely from IGN aerial orthophotographs, without drone support. This spatial incompleteness of drone coverage is one of the factors that motivated the choice of a uniform, institutionally maintained image source as model input. All polygon layers were digitised at a consistent, fixed scale of 1:144, matching the scale used by Le Meur [31] for the same estuarine sites; digitisation consistency across operators and sessions was ensured by enforcing this fixed reference scale rather than a variable, screen-zoom-dependent digitisation, following the same protocol as Le Meur [31]. All layers were integrated into QGIS under the Lambert-93 projection (EPSG:2154). This polygonal dataset serves as the reference labeling database used for training and validating the deep learning models.

Table 1 summarises, for each labelled site, the reference imagery used for labelling, the field or drone survey date, the labelling source, and a qualitative reference-quality tier (terrain survey > drone orthophotograph > IGN photo-interpretation), reflecting the expected positional accuracy of each source.

**Table 1:** Ground truth sources by labelled site. Reference quality tiers, from highest to lowest expected positional accuracy: terrain (GNSS/DGPS field survey), drone (centimetric photogrammetric orthophotograph), IGN photo-interpretation (digitisation from BD ORTHO^®^ without field or drone support).

Site	Reference imagery	Field/drone survey date	Labelling source	Quality tier
Daoulas estuary	2025 drone orthophotograph (eBee X / Mavic 3E)	14, 18, 19 Mar. 2025 (GNSS); 29 Apr. 2025 (drone)	GNSS field survey + drone photogrammetry	Terrain + drone
Mengleuz / Rosmélec	2025 drone orthophotograph (Mavic 3E)	29 Apr. 2025 (drone)	Drone photogrammetry	Drone
Le Faou	2025 drone orthophotograph	29 Apr. 2025 (drone)	Drone photogrammetry + field verification	Terrain + drone
Keroullé	IGN BD ORTHO^®^	Not applicable (no field/drone campaign at this site)	IGN photo-interpretation	Photo-interpretation
Le Pédel	IGN BD ORTHO^®^	Not applicable (no field/drone campaign at this site)	IGN photo-interpretation	Photo-interpretation
Pont-Callec	IGN BD ORTHO^®^	Not applicable (no field/drone campaign at this site)	IGN photo-interpretation	Photo-interpretation

For Le Pédel and Pont-Callec, historical vegetation surface data (1952–2004) originally produced by Sparfel et al. [29] using MapInfo were also recovered and integrated after conversion to shapefile format and reprojection to Lambert-93, contributing to the diachronic analysis but not to the patch-level training labels. *Limonium humile* individual stands were censused by DGPS during the 26–27 June 2025 field campaign but were recorded as point occurrences rather than digitised as polygons, owing to their small individual footprint relative to the labelling scale.

**Model input data and dataset construction.** Although the drone orthophotographs produced in 2025 offer a better spatial resolution for habitat delineation, the present study deliberately uses the IGN BD ORTHO^®^ CIR orthoimage (20 cm GSD) combined with a LiDAR HD-derived digital surface model (DSM, resampled to 20 cm GSD; IGN LiDAR HD national programme, Brittany acquisition campaign, winter 2024–2025) as exclusive model inputs. This choice is motivated by three complementary and explicitly stated considerations.

First, the operational objective of this study is to evaluate the feasibility of automating *Spartina* detection from routinely updated, institutionally maintained imagery. The BD ORTHO^®^ mission provides nationwide CIR coverage of metropolitan France on a regular update cycle at no acquisition cost for public institutions, and constitutes a realistic and sustainable operational baseline for coastal managers including the Parc Naturel Régional d’Armorique (PNRA) who cannot rely on dedicated drone campaigns for systematic annual monitoring.

Second, this study is designed as a **transferability framework**. Labels derived from high-accuracy drone and field data were co-registered and transferred onto the BD ORTHO^®^ grid, providing high-quality supervision signal for a lower-resolution input space. Training models on this coarser but operationally realistic imagery constitutes a methodological bridge towards other regularly updated spaceborne sensors at comparable or coarser resolutions such as Pléiades Néo (30–50 cm), SPOT-7 (1.5 m), or Sentinel-2 (10 m), for which revisit frequency and acquisition cost are considerably more favourable than dedicated drone campaigns. These sensors differ substantially from BD ORTHO^®^ in spatial resolution, spectral response, and acquisition geometry, so this extrapolation is not automatic: transferring the present pipeline to any of these sensors would require dedicated retraining and independent validation on sensor-specific data, which we identify as a priority direction for future work rather than a capability demonstrated here.

Third, working at 20 cm GSD on a **limited, imbalanced dataset** is a deliberate experimental choice that reflects the realistic data conditions faced by practitioners in coastal vegetation monitoring. It allows a rigorous evaluation of model and training strategy behaviour under the data scarcity and class imbalance constraints that are inherent to operational geospatial deep learning, where large-scale labelled datasets are rarely achievable.

The Digital Surface Model (DSM) used as the fourth input channel was obtained from the IGN LiDAR HD programme, which provides nationally consistent airborne LiDAR acquisitions at a minimum pulse density of 10 pts m^-2^. The DSM product (referred to as MNS – Modèle Numérique de Surface – in the IGN nomenclature) is distributed as a pre-processed raster derived directly from the classified LiDAR HD point cloud. It provides the absolute surface elevation of all above-ground elements, including terrain, low, medium and tall vegetation, buildings, and bridge decks, interpolated from the corresponding classified point classes onto a regular 50 cm grid in GeoTIFF format. Point cloud classification, ground filtering, and raster interpolation are performed entirely by IGN and validated against ground control points measured by IGN surveyors prior to public distribution [35]. In the intertidal context of this study, the absolute elevation encoded in the DSM is expected to carry ecologically meaningful information: *Spartina* colonisation in the Bay of Brest is restricted to a narrow tidal elevation window at the low-schorre margin, so that surface elevation acts as a proxy for tidal inundation regime and, in principle, helps to separate *Spartina* fronts, unvegetated mudflat, and mid-schorre halophytic communities [28]. The extent to which this expected contribution is actually realised by the segmentation model, given the way the DSM is combined with the spectral channels, is quantified empirically in Section 3.4 and discussed in Section 4.4. The DSM tiles were downloaded at their native resolution of 50 cm via the IGN web distribution service and resampled to 20 cm GSD using bilinear interpolation in QGIS to match the spatial resolution of the BD ORTHO^®^ CIR orthoimage, prior to co-registration and four-channel stack assembly.

The labelled vector polygons were rasterised and co-registered with the BD ORTHO^®^ CIR orthoimage and the LiDAR HD DSM to produce a four-channel raster stack (R, G, NIR, DSM) at 20 cm GSD. The dataset was then tiled into non-overlapping patches of 224×224×4 pixels, yielding a total of 810 patches. Each patch was assigned a binary segmentation mask (*Spartina* = 1; background = 0). The class distribution is moderately imbalanced, with *Spartina* pixels representing approximately 24.8% of the total labelled surface, a realistic figure for low-schorre intertidal environments in the Bay of Brest. The dataset was split into five stratified folds for cross-validation, ensuring that each fold preserves the spatial distribution of labelled patches across the study sites and maintains a balanced representation of the class imbalance across training and validation subsets.

**Spatial autocorrelation considerations.** A known source of bias in geospatial deep learning is spatial autocorrelation between training and validation samples, which can inflate performance metrics when nearby patches share similar spectral and structural properties [36,37]. In the present study, several design choices were implemented to mitigate this effect, and their combined impact was characterised quantitatively through a fold composition analysis and a patch-level topological assessment (Table 2).

**Table 2:** Composition of the five cross-validation folds and spatial autocorrelation characterisation. *Spartina* % refers to the proportion of positive pixels in the subset. Mean card. train nbrs: mean number of cardinal neighbours (N, S, E, W) of a validation patch belonging to the training subset (max. 4; expected ≈ 2.8 under random assignment). Fully surr. (%): percentage of validation patches entirely surrounded by training patches in all eight directions (expected ≈ 26% under random assignment; observed mean 34.4% indicates moderate spatial autocorrelation).

Fold	Subset composition				Validation patches per site					Spatial topology	
	*N*_train_	*N*_val_	*Spartina* %_train_	*Spartina* %_val_	Le Faou^c^	Daoulas	Le Pédel	Pont-Callec	Mengleuz	Mean card. train nbrs (/4)^a^	Fully surr. (%)^b^
0	648	162	24.4	26.4	66	49	21	13	13	2.41	35.2
1	648	162	25.1	23.6	79	48	10	13	12	2.28	29.6
2	648	162	25.4	22.4	61	57	16	15	13	2.39	33.3
3	648	162	24.3	26.7	60	63	17	11	11	2.46	40.1
4	648	162	24.8	24.9	75	46	14	13	14	2.36	34.0
**Mean**	648	162	24.8	24.8	68	53	16	13	13	2.38	34.4
**Std**	—	—	0.5	1.7	8	7	4	2	1	0.07	3.8

^a^Mean number of the four cardinal neighbours (N, S, E, W) of a validation patch that belong to the training subset. Maximum possible value: 4. Expected value under spatially random assignment: $0.80\times {\bar{n}}_{\text{card}}\approx 2.8$, where 0.80 is the training proportion (648/810) and ${\bar{n}}_{\text{card}}$ is the mean number of existing cardinal neighbours per patch on the irregular patch grid.

^b^Percentage of validation patches for which all existing neighbours (cardinal and diagonal, up to 8) belong to the training subset. Expected value under spatially random assignment: ≈26% (0.80^6^, assuming a mean of six existing neighbours). The observed mean of 34.4% indicates a moderate spatial autocorrelation exposure, intermediate between a strict spatial block split (0%) and a pixel-level random split (≈26%).

^c^This column aggregates the Le Faou estuary and the adjacent Keroullé inlet, which were treated as a single labelling unit in the fold composition analysis (283 and 58 patches respectively, 341 in total). The two are reported separately in the leave-one-site-out analysis (Section 3.5, Table 10), where site membership was reassigned from the georeferenced extent of each patch. All other columns are identical under both assignments.

The dataset was tiled into strictly non-overlapping patches extracted from a regular grid, ensuring that no pixel is shared between training and validation subsets within any fold. Labelled data were drawn from six geographically distinct estuarine zones (Daoulas, Mengleuz/Rosmélec, Le Faou, Keroullé, Le Pédel, Pont-Callec), each separated by several kilometres of rocky coastline and characterised by distinct morphological and ecological configurations (Fig 2). Five-fold cross-validation was employed to average performance estimates across multiple spatial partitions, thereby limiting the influence of any single spatial arrangement on the reported metrics.

The fold composition analysis confirms that the five folds are statistically comparable: the *Spartina* pixel ratio in the validation subset varies between 22.4% and 26.7% across folds (mean 24.8±1.7%), while the corresponding training ratio remains stable between 24.3% and 25.4% (mean 24.8±0.5%). All five study sites are represented in both the training and validation subsets of each fold, ensuring that no configuration deprives the model of an entire site during a given fold (Table 2).

To quantify the residual spatial autocorrelation exposure, a topological analysis was conducted for each fold: for each validation patch, the number of immediate cardinal neighbours (N, S, E, W) belonging to the training subset was computed from patch georeferenced centroids, along with the proportion of validation patches entirely surrounded by training patches across all eight directions. Averaged over the five folds, 2.38±0.07 of the four possible cardinal neighbours of a validation patch are training patches (59.5%), and 34.4±3.8% of validation patches are entirely surrounded by training patches. These values are intermediate between the two theoretical extremes: a strict spatial block split, in which validation patches would neighbour only other validation patches (0% surrounded), and a spatially random pixel-level assignment, under which each neighbour would be a training patch with probability 0.80 (648/810), yielding an expected surrounding rate of approximately 26% (0.80^6^, assuming a mean of six existing neighbours per patch). The observed mean of 34.4% is moderately above this random expectation, confirming the presence of residual spatial autocorrelation without extreme clustering. Critically, this topological exposure is stable across folds (29.6% to 40.1%), meaning that any resulting metric inflation applies uniformly across all 180 configurations evaluated under the same cross-validation protocol. The relative ranking of training strategies, which constitutes the central analytical object of this study, is therefore not compromised by this bias.

A strict block-level spatial split in which entire sites are withheld as validation folds was not adopted, as the limited number of sites and the uneven spatial distribution of *Spartina* across them would have produced folds with substantially imbalanced class ratios and insufficient sample sizes, undermining the statistical reliability of the ablation comparisons. An entirely independent test site (Hôpital-Camfrout) for which no labelled data entered the training process was used for operational generalisation assessment (Section 3.3), providing a spatially disjoint evaluation that is robust to autocorrelation by design. The trade-off between spatial independence and statistical power inherent to small geospatial datasets is further discussed in Section 4.5.

### 2.4. Segmentation architectures

All experiments were conducted using the SemanticSeg4EO framework [38,39], whose implementation and documentation are openly available on GitHub at https://github.com/aleguillou1/SemanticSeg4EO.

**Fig 3:**
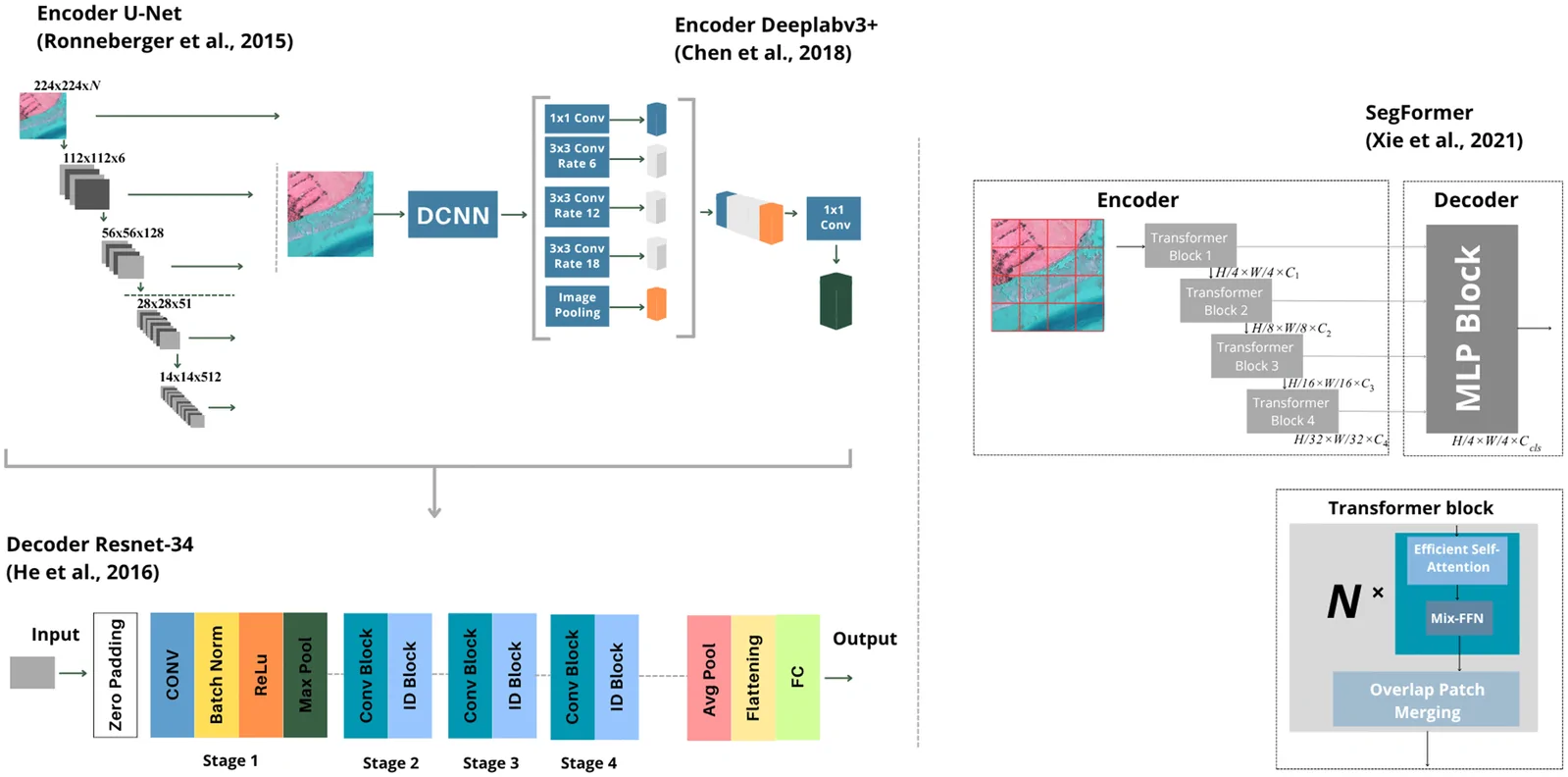
Architectural overview of the three segmentation models evaluated for *Spartina* detection. From left to right: U-Net with ResNet-34 encoder–decoder and skip connections; DeepLabV3+ with ResNet-34 and Atrous Spatial Pyramid Pooling; SegFormer-B2 with hierarchical Mix Transformer encoder and lightweight MLP decoder.

Three segmentation architectures were selected to represent the major structural paradigms in deep learning-based image segmentation, enabling a systematic evaluation of how architectural design influences model behaviour under small-dataset, spectrally complex conditions. The three families span the range from purely convolutional designs to attention-based Transformers: classical symmetric encoder–decoder networks (U-Net; [16]), dilated convolutional networks with multi-scale pooling (DeepLabV3 + ; [17]), and hierarchical Transformer architectures (SegFormer-B2; [18]). The CNN-based architectures share a ResNet-34 backbone [40] pretrained on ImageNet-1k, while SegFormer-B2 relies on a Mix Transformer (MiT-B2) encoder. Parameter counts are comparable across the three models (~24–26 M), ensuring that observed performance differences reflect architectural mechanisms rather than representational capacity (Fig 3).

**Symmetric encoder–decoder with skip connections: U-Net.** U-Net employs a symmetric encoder–decoder structure in which skip connections directly concatenate high-resolution feature maps from each encoder stage to the corresponding decoder level. This design preserves spatial detail across multiple scales and is particularly suited to boundary-sensitive detection tasks such as *Spartina* mapping, where accurate delineation of narrow colonisation fronts at the low-schorre margin is critical. The ResNet-34 backbone, which replaces the original contracting path, leverages residual learning to stabilise gradient propagation during fine-tuning, an important property under the limited training set available in this study (~24 M parameters).

**Multi-scale context with dilated convolutions: DeepLabV3 + .** DeepLabV3 + captures multi-scale contextual information through its Atrous Spatial Pyramid Pooling (ASPP) module, which applies parallel dilated convolutions at multiple receptive field sizes to the encoder output without reducing spatial resolution. This multi-scale representation is relevant for *Spartina* detection, as colonisation front widths vary considerably across estuarine configurations in the Bay of Brest, from narrow pioneer fringes at the ecotone with bare mudflat to extensive monospecific stands. A lightweight decoder combines ASPP features with low-level encoder outputs to refine segmentation boundaries progressively. The same ResNet-34 backbone used in U-Net serves as the feature extractor (~26 M parameters).

**Attention-based hierarchical Transformer: SegFormer-B2.** SegFormer replaces convolutional feature extraction with a hierarchical Mix Transformer (MiT) encoder in which efficient multi-head self-attention captures long-range spatial dependencies across the full patch extent. This global receptive field allows the model to jointly exploit the complementary information provided by the CIR spectral channels and the LiDAR-derived DSM, whose discriminative value for *Spartina* detection is conditioned on spatial context beyond the immediate neighbourhood of each pixel. Crucially for this study, the MiT-B2 encoder integrates internal regularisation mechanisms, namely attention dropout and stochastic depth, which reduce sensitivity to externally imposed regularisation and partially compensate for limited input diversity. Position information is encoded implicitly through mixed depthwise convolutions rather than positional embeddings, making the architecture robust to the variable patch content characteristic of intertidal remote sensing. The decoder consists of lightweight MLP layers aggregating features across encoder scales (~25 M parameters). The B2 variant was selected as a trade-off between representational capacity and training stability under limited data conditions.

### 2.5. Parametric training framework

To systematically characterise the behaviour of the three segmentation architectures under small-dataset conditions, we designed a one-factor-at-a-time (OFAT) ablation experiment in which one hyperparameter family is varied at a time while all others are held constant at a shared reference configuration (Table 3). Four training strategy axes were evaluated: data augmentation policy, loss function, regularisation scheme, and optimisation strategy. These axes were selected for their known influence on generalisation under data scarcity and class imbalance conditions. Each axis comprises two to five strategies, yielding 12 unique configurations applied to each of the three architectures under 5-fold stratified cross-validation, for a total of 12×3×5=180 training runs. The patch size of 224×224 pixels was selected as a standard trade-off between three competing constraints: (i) compatibility with the 224×224 input resolution of ImageNet-pretrained encoders (ResNet-34 and MiT-B2), which avoids architectural modifications; (ii) GPU memory budget (16 GB VRAM, batch size 8), which limits the maximum feasible patch size; and (iii) dataset size preservation, as larger patches would reduce the number of available training samples below statistically meaningful levels on this 810-patch dataset. The sensitivity of results to patch size is acknowledged as a limitation and constitutes a natural follow-up experiment (Table 4).

**Table 3:** Experimental design of the ablation study. The three segmentation architectures and four training strategy axes are summarised. For each axis, one hyperparameter family is varied while all others remain at the reference configuration (grey rows). Fixed shared parameters: patch size 224×224 px, batch size 8, learning rate 10^−4^, maximum 75 training epochs with early-stopping patience of 20 epochs. All configurations were evaluated by 5-fold cross-validation on 810 patches (CIR + DSM, 4 channels, 20 cm GSD), yielding 12×3×5=180 training runs.

Component	Strategy / Level	Description
**Segmentation architecture**	U-Net	Encoder–decoder with skip connections (CNN); ResNet-34 backbone; ~24 M parameters
	DeepLabV3+	Dilated convolutions + ASPP pooling (CNN); ResNet-34 backbone; ~26 M parameters
	SegFormer-B2	Hierarchical Transformer with efficient self-attention; MiT-B2 encoder; ~25 M parameters
**Fixed parameters**	Pre-training	ImageNet-1k for all encoders (ResNet-34 and MiT-B2)
	Input	4 channels: R, G, NIR (CIR orthoimage) + DSM
**A. Data augmentation**	None	No augmentation applied during training
	**Basic (ref.)**	Horizontal/vertical flip, random rotation (±45°), brightness and contrast jitter
	Advanced	Basic + elastic deformation, grid distortion, random crop
	Aggressive	Advanced + Gaussian noise, Gaussian blur, coarse dropout (20%)
	Extreme	Aggressive + channel shuffle, hue shift, heavy elastic distortion
**B. Loss function**	BCE	Binary cross-entropy: $-[y\log\hat{p}+(1-y)\log(1-\hat{p})]$
	**Dice+BCE (ref.)**	$ℒ=0.5\;{ℒ}_{Dice}+0.5\;{ℒ}_{BCE}$
	Focal+Dice	$ℒ=0.5\;{ℒ}_{Focal}(\gamma =2)+0.5\;{ℒ}_{Dice}$
	Tversky	${ℒ}_{Tv}(\alpha =0.3,\;\beta =0.7)$; asymmetric weighting penalising false negatives
**C. Regularisation**	Dropout = 0.0	No dropout on the decoder head
	**Dropout = 0.2 (ref.)**	Dropout applied to the decoder head (*p* = 0.2)
	Dropout = 0.5	Dropout applied to the decoder head (*p* = 0.5)
	Class weights	Loss weighted by inverse class frequency ($w_{spartina}\approx 3.3$)
**D. Optimisation**	**Cosine annealing (ref.)**	AdamW, $T_{\max}=75$, $\eta_{\min}=10^{-6}$; full encoder fine-tuning
	Freeze + Warmup	Encoder frozen for 10 epochs then unfrozen; cosine annealing schedule
	ReduceOnPlateau	AdamW, reduction factor 0.5, patience 5; full encoder fine-tuning

**Table 4:** Complementary ablation results: F1-score, Precision, Recall and Overall Accuracy (%) ± std over 5-fold cross-validation. Bold: best per axis per architecture. Underline: worst.

Axis	Strategy	F1-score (%)			Precision (%)		
		U-Net	DLV3+	SF-B2	U-Net	DLV3+	SF-B2
A. Data augmentation	None	82.6 ± 1.5	82.7 ± 1.3	85.5 ± 1.0	83.7 ± 3.5	82.8 ± 2.7	84.5 ± 1.5
	Basic (ref)	85.6 ± 1.0	**85.7 ± 1.0**	**85.9 ± 1.5**	84.3 ± 2.0	**84.9 ± 1.5**	84.8 ± 2.0
	Advanced	**86.0 ± 1.1**	85.6 ± 1.0	85.8 ± 1.2	**86.0 ± 1.8**	84.0 ± 1.5	**85.3 ± 1.7**
	Aggressive	84.5 ± 1.2	84.5 ± 1.4	85.2 ± 1.7	84.4 ± 1.8	83.8 ± 1.7	83.8 ± 2.5
	Extreme	83.8 ± 1.1	83.4 ± 1.2	84.9 ± 1.9	82.7 ± 1.6	81.6 ± 1.0	83.2 ± 2.2
B. Loss function	BCE	**85.9 ± 1.4**	**85.7 ± 1.3**	**86.3 ± 1.4**	**85.1 ± 1.9**	**85.8 ± 1.2**	**86.1 ± 1.5**
	Dice+BCE (ref)	85.6 ± 1.0	85.7 ± 1.0	85.9 ± 1.5	84.3 ± 2.0	84.9 ± 1.5	84.8 ± 2.0
	Focal+Dice	85.8 ± 1.1	85.0 ± 1.2	85.9 ± 1.2	84.4 ± 0.5	83.0 ± 1.6	85.1 ± 1.1
	Tversky	82.7 ± 1.6	82.5 ± 1.1	82.8 ± 1.2	76.0 ± 2.5	75.6 ± 1.7	76.2 ± 2.7
C. Regulari- sation	Dropout = 0.0	85.5 ± 1.1	85.6 ± 1.1	**86.2 ± 1.6**	**86.0 ± 1.4**	84.8 ± 1.2	**85.9 ± 2.2**
	Dropout = 0.2 (ref)	85.6 ± 1.0	**85.7 ± 1.0**	85.9 ± 1.5	84.3 ± 2.0	**84.9 ± 1.5**	84.8 ± 2.0
	Dropout = 0.5	85.6 ± 1.4	85.4 ± 1.1	82.0 ± 1.5	84.4 ± 2.0	83.9 ± 1.6	80.1 ± 2.5
	Class weights	**86.1 ± 1.0**	85.5 ± 1.1	86.0 ± 1.7	85.5 ± 1.5	84.1 ± 1.4	84.1 ± 2.2
D. Optimi- sation	Cosine (ref)	85.6 ± 1.0	**85.7 ± 1.0**	**85.9 ± 1.5**	84.3 ± 2.0	**84.9 ± 1.5**	**84.8 ± 2.0**
	Freeze + Warmup	78.5 ± 0.9	78.9 ± 1.2	81.7 ± 1.3	76.9 ± 1.2	77.2 ± 3.1	77.9 ± 1.8
	ReduceOnPlateau	**85.6 ± 1.2**	85.6 ± 1.1	85.6 ± 1.7	**85.5 ± 1.1**	84.6 ± 1.6	84.7 ± 1.9

All architectures share a fixed baseline: ImageNet-1k pretrained encoders, 224×224×4 patches (R, G, NIR, DSM) at 20 cm GSD, batch size 8, learning rate 10^−4^, a maximum of 75 training epochs, and early stopping with a patience of 20 epochs (monitored on validation IoU). The reference configuration: Basic augmentation, Dice+BCE, Dropout = 0.2, Cosine annealing with full encoder fine-tuning serves as the common point of comparison across all axes (Table 5).

**Table 5:** Complementary ablation results (continued): Recall and Overall Accuracy (%) ± std over 5-fold cross-validation. Bold: best per axis per architecture. Underline: worst.

Axis	Strategy	Recall (%)			OA (%)		
		U-Net	DLV3+	SF-B2	U-Net	DLV3+	SF-B2
A. Data augmentation	None	81.7 ± 1.1	82.7 ± 1.4	86.4 ± 1.0	91.5 ± 0.4	91.5 ± 0.2	92.7 ± 0.6
	Basic (ref)	**87.0 ± 0.6**	86.5 ± 0.9	**87.0 ± 1.5**	92.8 ± 0.2	**92.9 ± 0.4**	**92.9 ± 0.5**
	Advanced	86.0 ± 1.2	**87.2 ± 0.6**	86.4 ± 1.8	**93.1 ± 0.4**	92.7 ± 0.4	92.9 ± 0.5
	Aggressive	84.6 ± 0.7	85.2 ± 2.2	86.7 ± 1.4	92.4 ± 0.3	92.3 ± 0.4	92.6 ± 0.7
	Extreme	85.0 ± 0.8	85.3 ± 1.8	86.8 ± 2.0	91.9 ± 0.2	91.6 ± 0.3	92.4 ± 0.7
B. Loss function	BCE	86.6 ± 1.1	85.6 ± 1.6	86.4 ± 1.5	**93.0 ± 0.4**	**93.0 ± 0.4**	**93.2 ± 0.6**
	Dice+BCE (ref)	87.0 ± 0.6	86.6 ± 0.9	87.0 ± 1.5	92.8 ± 0.2	92.9 ± 0.4	92.9 ± 0.5
	Focal+Dice	87.3 ± 1.7	87.1 ± 0.9	86.7 ± 1.7	92.9 ± 0.2	92.4 ± 0.5	93.0 ± 0.4
	Tversky	**90.8 ± 1.7**	**90.7 ± 1.1**	**90.7 ± 1.7**	90.6 ± 0.8	90.5 ± 0.5	90.6 ± 0.7
C. Regulari- sation	Dropout = 0.0	85.1 ± 1.1	86.4 ± 1.4	86.5 ± 1.1	92.9 ± 0.4	92.8 ± 0.3	**93.2 ± 0.7**
	Dropout = 0.2 (ref)	**87.0 ± 0.6**	86.6 ± 0.9	87.0 ± 1.5	92.8 ± 0.2	**92.9 ± 0.4**	92.9 ± 0.5
	Dropout = 0.5	87.0 ± 1.1	**87.0 ± 0.9**	83.9 ± 1.0	92.8 ± 0.4	92.7 ± 0.5	90.9 ± 0.7
	Class weights	86.6 ± 1.1	87.0 ± 1.2	**88.0 ± 1.9**	**93.1 ± 0.4**	92.7 ± 0.4	92.9 ± 0.7
D. Optimi- sation	Cosine (ref)	**87.0 ± 0.6**	86.5 ± 0.9	**87.0 ± 1.5**	92.8 ± 0.2	**92.9 ± 0.4**	**92.9 ± 0.5**
	Freeze + Warmup	80.2 ± 1.3	80.8 ± 1.8	85.9 ± 1.3	89.1 ± 0.4	89.3 ± 0.5	90.5 ± 0.2
	ReduceOnPlateau	85.7 ± 1.4	**86.7 ± 0.7**	86.4 ± 1.8	**92.9 ± 0.3**	92.8 ± 0.4	92.8 ± 0.7

DLV3+ = DeepLabV3 + , SF-B2 = SegFormer-B2. All models use a ResNet-34 encoder with ImageNet-pretrained weights.

Augmentation levels were designed as a graduated series from no transformation to increasingly disruptive geometric and photometric perturbations, in order to identify the saturation point beyond which additional diversity degrades learning on this small dataset. The Tversky loss (α=0.3, β=0.7) was included specifically to test empirically whether the moderate class imbalance (~24.8% *Spartina* pixels) justifies its recall-oriented asymmetric weighting over simpler composite losses. The regularisation axis is of particular interest given the structural differences between CNN and Transformer decoders, which incorporate distinct internal regularisation mechanisms and may therefore respond differently to externally imposed dropout. Finally, the Freeze  +  Warmup optimisation strategy was included to assess whether this common transfer learning heuristic is beneficial or detrimental when the source domain (ImageNet RGB) and target domain (CIR  +  DSM) are substantially different.

### 2.6. Statistical analysis

All statistical analyses were performed in Python 3.12.13, using NumPy 2.4.4, pandas 3.0.3, SciPy 1.18.0, and scikit-learn 1.9.0 (for the five-fold partitioning). Deep learning models were implemented in PyTorch 2.12.1 and torchvision 0.27.1; U-Net and DeepLabV3 + were built on the segmentation_models_pytorch library (v0.5.0). The patch-to-site spatial assignment used for the leave-one-site-out validation (Section 3.5) used GeoPandas 1.1.4. All analysis code is openly available on GitHub (Section 2.4).

For each of the four training-strategy axes, differences in IoU across configurations were tested, within each architecture, using the Friedman test (scipy.stats.friedmanchisquare), a non-parametric test for repeated measures appropriate to the paired, non-normally distributed nature of five-fold cross-validation scores. Because twelve such tests were conducted simultaneously (four axes × three architectures), resulting *p*-values were adjusted for the false discovery rate using the Benjamini–Hochberg step-up procedure [41] at *q* = 0.05; all significance statements in this article refer to these adjusted values ($p_{adj}$) unless stated otherwise, and the threshold for significance was set at α=0.05 throughout. Effect sizes are reported as paired Cohen’s $d_{z}$: for each configuration, the mean of the per-fold differences in IoU relative to the reference configuration (same five-fold partition) was divided by the standard deviation of those differences (with Bessel’s correction). No imputation was required, as no data were missing from any fold; outlier folds were not excluded from any analysis, and all five folds contribute to every reported mean and test regardless of their individual performance.

## 3. Results

### 3.1. Ablation study: Impact of training strategies across segmentation architectures

**Fig 4:**
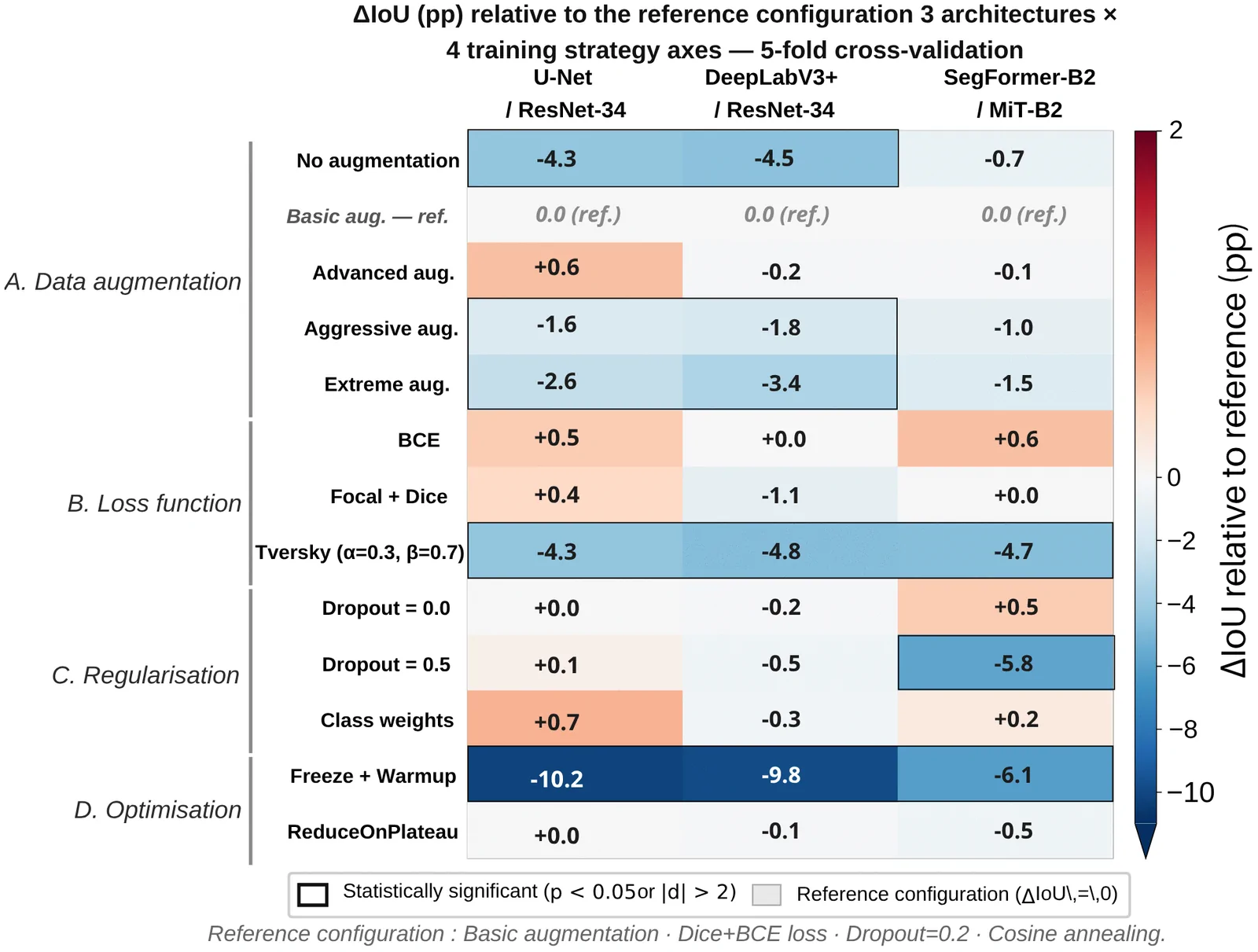
Ablation study results: ΔIoU (pp) relative to the reference configuration. ΔIoU across three segmentation architectures and four training strategy axes. Black borders indicate a statistically significant effect after correction for multiple comparisons (Friedman $p_{adj}<0.05$). Effect sizes (*d*; paired Cohen’s $d_{z}$, defined in Section 2.6) are reported separately in the main text for configurations exceeding a conventional large-effect threshold (|*d*| > 2); statistical significance and effect magnitude are distinct properties and are not conflated here. Grey row: reference configuration (ΔIoU = 0 by definition).

Table 3 and Fig 4 summarise the experimental design: twelve training configurations spanning four axes (data augmentation, loss function, regularisation, and optimisation strategy) were evaluated on three architectures U-Net with ResNet-34, DeepLabV3+ with ResNet-34, and SegFormer-B2 each trained with five-fold cross-validation on 810 patches of 224×224 pixels (CIR orthoimage + DSM, 20 cm GSD), yielding 180 training runs in total. The full quantitative results are reported in Table 6; the sensitivity heatmap (Fig 4) provides a visual synthesis of which strategies induce significant degradation per architecture. Four main findings emerge from this systematic comparison.

**Table 6:** Ablation study results: mean IoU (%) ± std over 5-fold cross-validation. Bold: best per axis per architecture. Underline: worst. $p_{adj}$ values are Friedman test *p*-values adjusted for multiple comparisons using the Benjamini–Hochberg false discovery rate procedure (*m* = 12 tests; *q* = 0.05); †: significant after correction. No previously significant effect became non-significant after adjustment.

Axis	Strategy	U-Net	DeepLabV3+	SegFormer-B2
A. Data augmentation	None	70.5 ± 2.2	70.5 ± 1.9	74.6 ± 1.5
	Basic (ref)	74.8 ± 1.5	**75.0 ± 1.6**	**75.3 ± 2.3**
	Advanced	**75.4 ± 1.7**	74.8 ± 1.5	75.2 ± 1.9
	Aggressive	73.2 ± 1.8	73.2 ± 2.1	74.3 ± 2.5
	Extreme	72.2 ± 1.6	71.6 ± 1.8	73.8 ± 2.8
	$p_{adj}$ ^a^	$0.003^{†}$	$0.003^{†}$	0.193
B. Loss function	BCE	**75.3 ± 2.2**	**75.0 ± 2.0**	**75.9 ± 2.1**
	Dice+BCE (ref)	74.8 ± 1.5	75.0 ± 1.6	75.3 ± 2.3
	Focal+Dice	75.2 ± 1.7	73.9 ± 1.8	75.3 ± 1.9
	Tversky	70.5 ± 2.3	70.2 ± 1.5	70.6 ± 1.7
	$p_{adj}$ ^a^	$0.033^{†}$	$0.016^{†}$	$0.035^{†}$
C. Regulari- sation	Dropout = 0.0	74.8 ± 1.8	74.8 ± 1.8	**75.8 ± 2.5**
	Dropout = 0.2 (ref)	74.8 ± 1.5	**75.0 ± 1.6**	75.3 ± 2.3
	Dropout = 0.5	74.9 ± 2.2	74.5 ± 1.6	69.5 ± 2.1
	Class weights	**75.5 ± 1.6**	74.7 ± 1.7	75.5 ± 2.6
	$p_{adj}$ ^a^	0.388	0.392	$0.035^{†}$
D. Optimi- sation	Cosine (ref)	74.8 ± 1.5	**75.0 ± 1.6**	**75.3 ± 2.3**
	Freeze + Warmup	64.6 ± 1.2	65.2 ± 1.7	69.1 ± 1.8
	ReduceOnPlateau	**74.8 ± 1.8**	74.9 ± 1.6	74.8 ± 2.6
	$p_{adj}$ ^a^	$0.035^{†}$	$0.035^{†}$	$0.035^{†}$

^a^Benjamini–Hochberg adjusted *p*-values (*q* = 0.05, *m* = 12 simultaneous Friedman tests). Values shown are computed from the *p*-values reported in this study.

*Reference configuration* (grey rows in Table 3): pretrained encoder (ResNet-34 / MiT-B2), basic augmentation, Dice+BCE loss, dropout = 0.2, cosine annealing, lr = 10^−4^, batch = 8.

Statistical significance was assessed using the Friedman test with Benjamini–Hochberg correction for the twelve simultaneous comparisons, and effect magnitude using paired Cohen’s $d_{z}$, as detailed in Section 2.6. All significance statements below refer to Benjamini–Hochberg adjusted *p*-values ($p_{adj}$) unless stated otherwise.

Because $d_{z}$ is computed from only five paired observations, a small standard deviation of the paired differences can yield a large standardised effect even when the underlying IoU gap is on the order of a few percentage points; several effect sizes reported below therefore exceed the conventional large-effect threshold (|*d*| > 2) despite comparatively modest raw score differences. Statistical significance and effect magnitude are accordingly reported and interpreted as distinct, complementary pieces of evidence throughout this study, rather than as equivalent indicators of the same property.

**Data augmentation.** Augmentation exerts a strong, architecture-dependent influence. For the two CNN-based models, the complete absence of augmentation leads to a substantial performance drop relative to the basic reference configuration (U-Net: −4.3 pts IoU, *d* = 4.0; DeepLabV3 + : −4.5 pts, *d* = 3.1; Friedman *p* < 0.001 for both), whereas SegFormer-B2 remains essentially unaffected (Δ=−0.7 pts, *p* = 0.161). This contrast is consistent with the hypothesis that SegFormer’s multi-scale self-attention mechanism acts as an implicit regularizer that compensates for reduced input diversity, a practically relevant property in small-dataset remote sensing contexts. Conversely, aggressive and extreme augmentation consistently harms all three architectures (U-Net: −2.6 pts from Basic to Extreme), confirming that excessive geometric distortions introduce noise that impairs feature learning when training data are limited. U-Net shows a marginal additional gain with *Advanced* augmentation over *Basic* (+0.6 pts, *d* = 0.7), whereas DeepLabV3 + plateaus at the *Basic* level suggesting a lower saturation threshold for dilated-convolution architectures.

**Loss function.** The loss function axis yields the most architecturally consistent result of the study. The Tversky loss (α=0.3, β=0.7) significantly degrades all three models (−4.3 to −4.8 pts IoU; Friedman p≤0.026 across all architectures), while BCE, Dice+BCE, and Focal+Dice are statistically interchangeable (Δ < 1 pt). This outcome is counter-intuitive given Tversky’s common recommendation for imbalanced segmentation tasks. However, with *Spartina* covering approximately 24.8% of pixels, the class imbalance appears insufficient to justify an asymmetric recall-oriented weighting, and the chosen hyperparameters (α=0.3, β=0.7) seem ill-calibrated for this configuration. This result has a direct practical implication: standard composite losses are preferable to specialised imbalance-correcting losses in near-balanced binary coastal vegetation mapping. Among these, Dice+BCE was retained as the reference configuration over BCE alone despite a marginally lower mean IoU (−0.1 to −0.6 pts, non-significant): its lower inter-fold variance on U-Net (±1.5% vs. ±2.2% for BCE) and its balanced precision–recall profile – BCE systematically inflates precision at the expense of recall under the present class distribution – make it the more robust choice for surface area estimation (Table 4).

**Regularisation.** The regularisation axis reveals the most pronounced architectural specificity in the entire experiment. U-Net and DeepLabV3 + are insensitive to dropout variations across the full range tested (Friedman *p* > 0.35 for both), whereas SegFormer-B2 is severely impacted by dropout = 0.5 (−5.8 pts IoU, *d* = 2.2, *p* = 0.026). This behaviour is consistent with SegFormer’s internal regularisation mechanisms attention dropout and stochastic depth embedded within the MiT-B2 encoder which, when combined with an additional external dropout layer, produce over-regularisation. Classic CNN architectures lack these internal mechanisms and therefore tolerate external dropout without performance loss. Class weights provide a marginal benefit for U-Net (Δ≈+0.7 pts) but have no meaningful effect on DeepLabV3+ or SegFormer-B2.

**Optimisation strategy.** Encoder freezing with warm-up produces the largest single effect observed across the entire ablation: catastrophic performance drops affect all three architectures simultaneously (U-Net: −10.2 pts, *d* = 11.4; DeepLabV3 + : −9.8 pts, *d* = 10.1; SegFormer-B2: −6.1 pts, *d* = 5.1; Friedman p≤0.022). With only 810 training patches acquired in the CIR + DSM domain substantially different from the RGB ImageNet pre-training distribution, full end-to-end fine-tuning of the encoder is indispensable. Pre-trained features do not transfer sufficiently under frozen-encoder conditions in this domain-shift context. By contrast, cosine annealing and ReduceOnPlateau schedulers yield equivalent results across all three architectures, indicating that the choice of learning-rate schedule is secondary once full fine-tuning is enabled.

**Fig 5:**
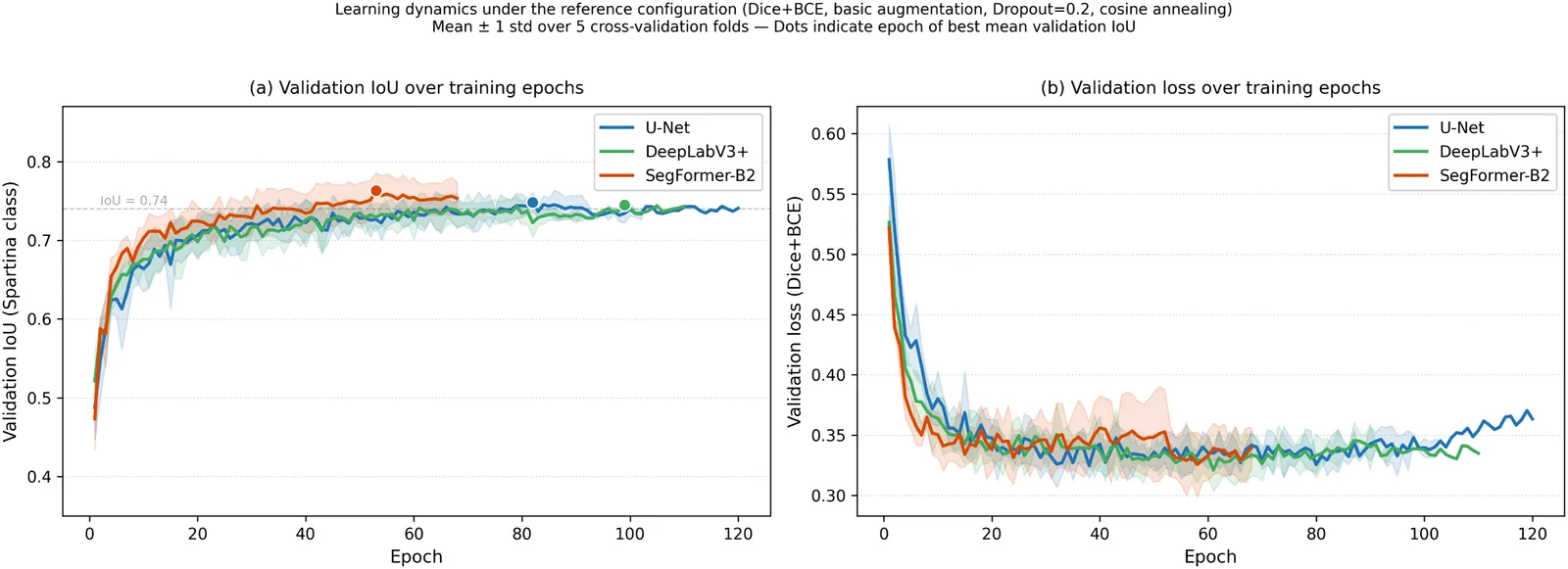
Learning dynamics of the three segmentation architectures under the reference training configuration. Dice+BCE loss, basic augmentation, Dropout = 0.2, cosine annealing schedule with full encoder fine-tuning. Each curve represents the mean validation IoU (panel a) and validation loss (panel b) over 5-fold cross-validation; shaded bands indicate ±1 standard deviation across folds. Dots mark the epoch of best mean validation IoU per architecture. The absence of systematic validation loss increase after convergence confirms that no severe overfitting occurs under these conditions, despite the limited dataset size (810 patches).

**Cross-architecture comparison.** Under the shared reference configuration, the three architectures achieve remarkably similar performance: U-Net 74.8±1.5%, DeepLabV3+ 75.0±1.6%, and SegFormer-B2 75.3±2.3% IoU, with a maximum spread of 0.5 percentage points (Table 4). The sensitivity heatmap (Fig 4) further confirms this convergence: only two strategies induce universal degradation across all three families (Tversky loss and encoder freezing), while architecture-specific vulnerabilities are limited to a single case (high dropout in SegFormer-B2). This constitutes a central finding of the study: **in this small-dataset, domain-specific remote sensing context, the choice of training strategy matters far more than the choice of segmentation architecture.** This disproportion between the strategy-induced range (~10 pts IoU) and the architectural spread (<1 pt) constitutes the central quantitative finding of the study, with direct implications for practitioners who invest effort in architectural selection rather than training pipeline design when developing similar automated mapping pipelines for coastal invasive vegetation. Fig 5 presents the validation IoU and loss trajectories over training epochs for the three architectures under the reference configuration. All models converge smoothly without systematic validation loss increase, confirming the absence of severe overfitting under the 810-patch training regime.

**Fig 6:**
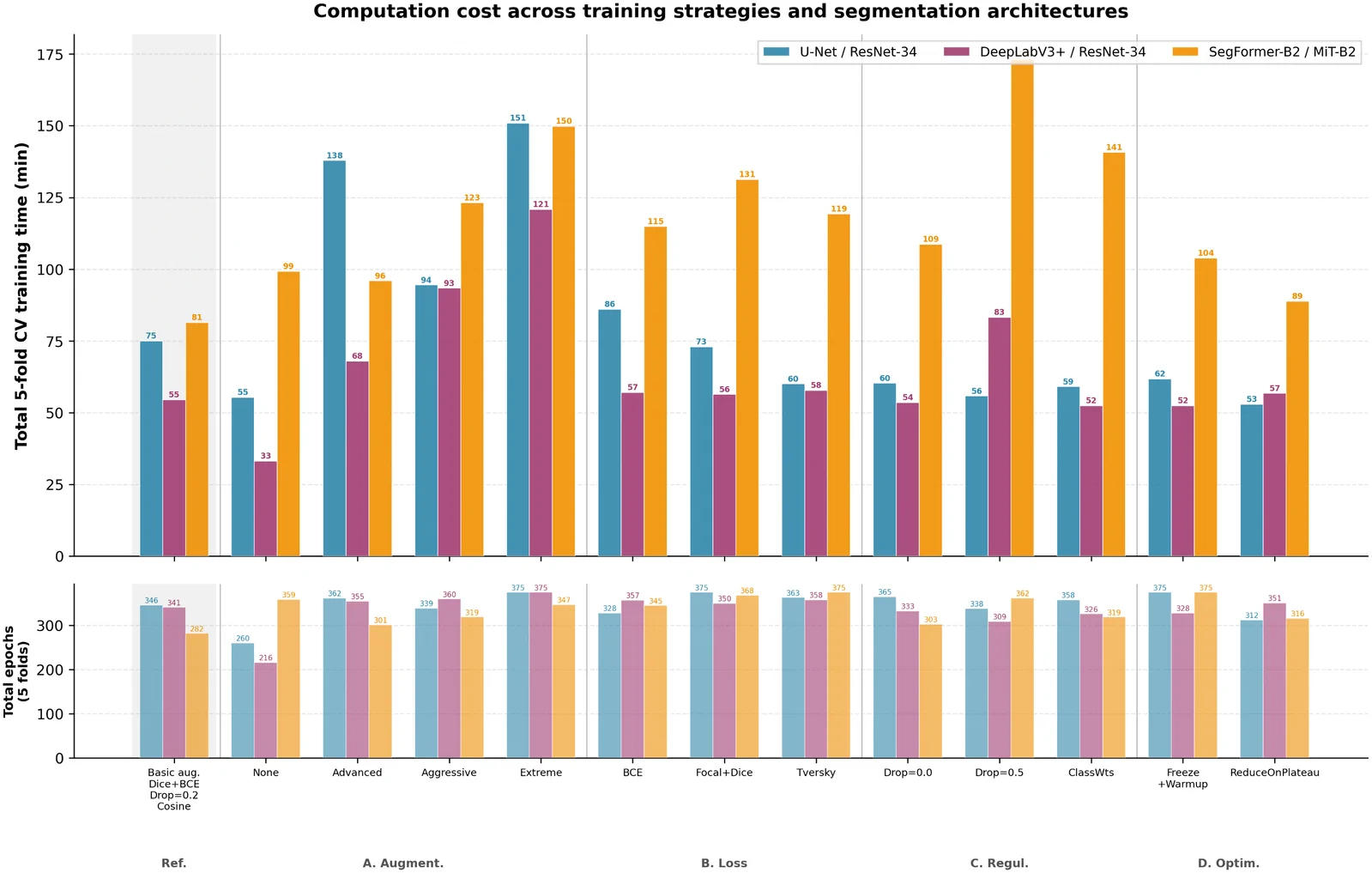
Comparison of total five-fold cross-validation training time and cumulative training epochs. Top panel: training time in minutes; bottom panel: cumulative number of training epochs across three segmentation architectures and twelve training strategy configurations. The reference configuration is highlighted in grey. Vertical lines separate the four ablation axes. Per-epoch cost is driven by architectural complexity (SegFormer-B2 > U-Net > DeepLabV3+), while total training time is primarily modulated by augmentation intensity and convergence dynamics.

Fig 6 reports the total five-fold cross-validation training time for each configuration and architecture. Two patterns emerge. The per-epoch cost differs markedly between architectural families: DeepLabV3 + is the most efficient (~9.6 s/epoch under the reference configuration), followed by U-Net (~13.0 s/epoch), while SegFormer-B2 is approximately 1.8× slower than DeepLabV3+ (~17.3 s/epoch), reflecting the higher computational overhead of multi-head self-attention relative to standard and dilated convolutions. Training strategy choices modulate computation time primarily through their effect on convergence speed rather than per-epoch cost. Augmentation complexity is the dominant time driver: *Extreme* augmentation doubles the total training time relative to *None* across all three architectures (U-Net: 151 vs. 55 min; DeepLabV3 + : 121 vs. 33 min; SegFormer-B2: 150 vs. 99 min), both because heavy geometric transforms increase per-epoch cost and because early stopping is triggered later (375 vs. 216–260 cumulative epochs summed across the five folds, out of a per-fold maximum of 75 epochs each; see Table 3). Loss function and regularisation axes, by contrast, have a limited effect on total time for the CNN architectures (<15% variation), but SegFormer-B2 exhibits a notable sensitivity: dropout  =  0.5 inflates its training time to 173 min the highest value across the entire experiment because the over-regularised model converges slowly without triggering early stopping. The Freeze  +  Warmup strategy, despite producing the worst segmentation performance, does not reduce computation: all five folds run to their individual maximum of 75 epochs for U-Net and SegFormer-B2 (375 cumulative epochs), indicating that frozen encoders prevent the model from reaching convergence rather than accelerating it. These results reinforce the practical recommendation that training strategy should be optimised not only for segmentation accuracy but also for computational budget, particularly when deploying pipelines on resource-constrained infrastructure typical of coastal management institutions.

### 3.2. Qualitative evaluation of the different semantic segmentation models

**Fig 7:**
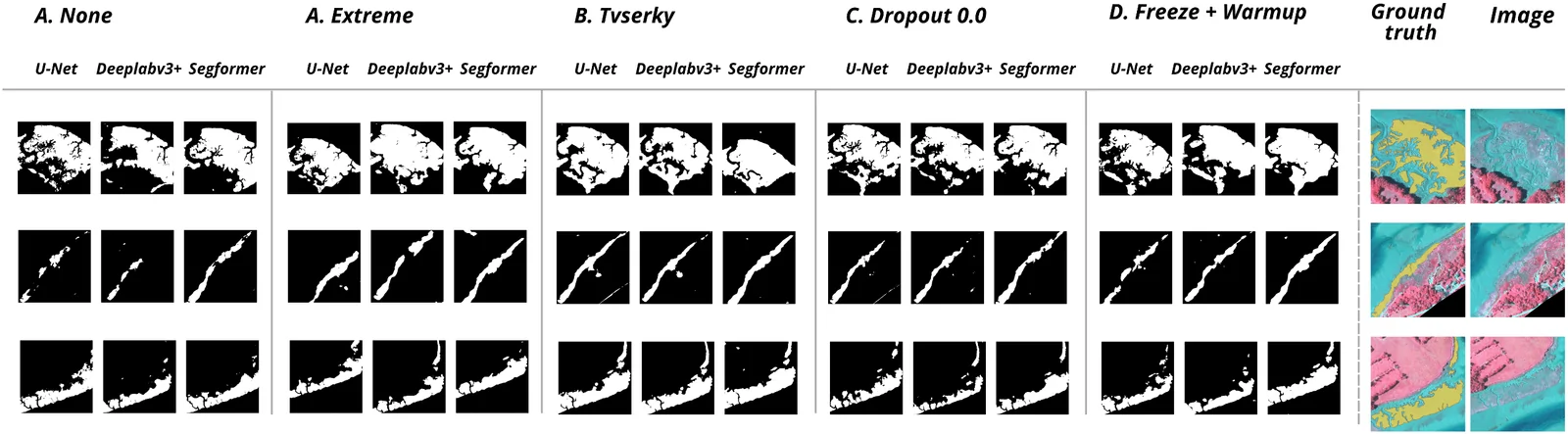
Qualitative comparison of segmentation masks across three ecologically distinct intertidal zones. Zones are within the Hôpital-Camfrout independent test site. Each zone presents a different configuration of Spartina colonisation relative to adjacent halophytic assemblages and bare mudflat. The figure illustrates the inference performance of U-Net (ResNet34), DeepLabv3+ (ResNet34), and SegFormer architectures under various training strategies (A–D) compared against the ground truth and original imagery.

A closer examination of the qualitative predictions reveals subtle but consistent architectural biases that persist across training strategies (Fig 7). SegFormer tends to produce spatially coherent predictions with fewer false positives, localising *Spartina* stands accurately at the patch level; however, it yields coarser boundary delineations, smoothing out fine-scale morphological details that carry cartographic significance. Conversely, U-Net generates sharper and more detailed segmentation boundaries, an advantage for mapping purposes but at the cost of lower detection sensitivity, particularly for small isolated rhizome clusters and narrow *Spartina* fringes at the ecotone between saltmarsh communities and bare mudflat. DeepLabV3 + exhibits intermediate behaviour, balancing boundary precision and detection completeness without clearly excelling at either. These architectural tendencies are consistent with the structural properties of each model: the global self-attention mechanism of SegFormer favours spatial regularity, whereas the skip connections and high-resolution feature propagation in U-Net preserve fine local detail at the expense of broader contextual coherence.

**Fig 8:**
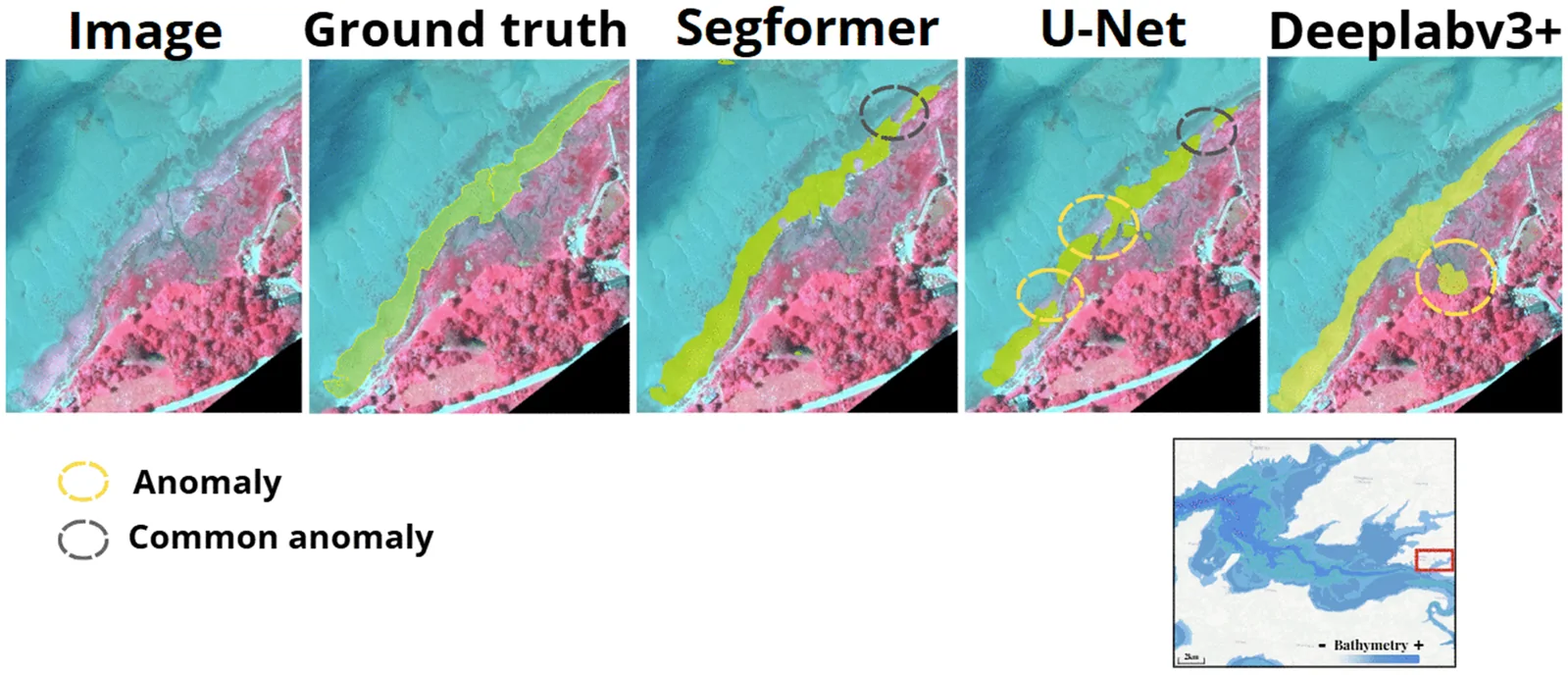
Qualitative comparison of segmentation predictions on an independent test zone. Each row displays the BD ORTHO^®^ CIR input image, the corresponding ground truth mask, and the predicted masks for SegFormer-B2, U-Net (ResNet-34), and DeepLabV3+ (ResNet-34), highlighting differences in boundary delineation, false positive rates, and detection sensitivity for isolated *Spartina* patches and mixed halophyte assemblages. Input imagery: IGN BD ORTHO^®^, Licence Ouverte / Etalab 2.0.

Despite these generally encouraging results, several limitations remain in the detection of small or mixed *Spartina* patches. The models perform reliably when *Spartina* stands are established on bare mudflat, the primary colonisation front on the low schorre where the spectral and structural contrast with the surrounding substrate is maximal. However, classification errors increase markedly in transitional zones where *Spartina* intermingles with *Halimione portulacoides* (at the distal end of tidal channels) or *Limonium vulgare* (on the upper mudflat fringe). In these mixed assemblages, even expert photo-interpreters struggle to delineate species boundaries with confidence, and the 20 cm GSD of the BD ORTHO^®^ imagery is likely insufficient to resolve the fine spectral and textural differences between these co-occurring halophytes. Future work could address this limitation through the integration of hyperspectral data or by adopting a multi-class segmentation framework that explicitly models the mixed *Spartina*–*Halimione*–*Limonium* continuum rather than treating it as a binary detection problem (Fig 7 and Fig 8).

### 3.3. Operational generalization and *Spartina* surface estimation across the Bay of Brest

Based on the ablation findings, the SegFormer-B2 architecture was retrained under the selected operational configuration (basic augmentation, Dice+BCE loss, dropout 0.2, cosine annealing with full encoder fine-tuning) with an extended training budget of 150 maximum epochs and a relaxed early stopping patience of 20. Five-fold cross-validation results are reported in Table 7.

**Table 7:** Five-fold cross-validation results of the SegFormer-B2 model under the selected operational configuration. Basic augmentation, Dice+BCE loss, dropout 0.2, cosine annealing, 150 max. epochs, patience 20.

Metric	Fold 0	Fold 1	Fold 2	Fold 3	Fold 4	Mean ± Std
IoU (%)	79.3	75.7	75.4	79.1	74.1	76.7 ± 2.1
F1-score (%)	88.5	86.2	86.0	88.3	85.1	86.8 ± 1.3
Precision (%)	88.2	84.9	86.1	85.5	85.0	85.9 ± 1.2
Recall (%)	88.7	87.4	85.9	91.2	85.3	87.7 ± 2.2
OA (%)	93.9	93.4	93.7	93.5	92.6	93.4 ± 0.5
Val. loss	0.326	0.311	0.313	0.304	0.316	0.314 ± 0.007
Epochs	117	116	60	86	77	91 ± 22
*95% confidence intervals*						
IoU 95% CI						[73.8, 79.6]
F1 95% CI						[84.9, 88.7]

The model achieved a mean IoU of 76.7±2.1% (95% CI: [73.8, 79.6]) and a mean F1-score of 86.8±1.3% (95% CI: [84.9, 88.7]), with an overall accuracy of 93.4±0.5% (Table 7). These results represent a consistent improvement of +1.4 pts IoU over the ablation reference (75.3±2.3%), attributable to the relaxed early stopping criterion (patience 20) which allows the model to converge more fully on the small training set. Per-fold IoU ranged from 74.1% (Fold 4) to 79.3% (Fold 0), with corresponding training durations between 60 and 117 epochs, reflecting variable convergence rates across spatial subsets of the study area. The low inter-fold standard deviation (±2.1 pts) and the narrow 95% confidence interval (±2.9 pts) confirm that the model generalises consistently across the different intertidal configurations represented in the cross-validation folds.

The precision–recall balance reported in Table 7 is of particular significance for surface area estimation. The model achieves a mean precision of 85.9±1.2% and a mean recall of 87.7±2.2%, yielding a near-symmetric error profile in which false positives (commission errors) and false negatives (omission errors) approximately compensate at the landscape scale. This balance implies that the model neither systematically overestimates nor underestimates colonised surfaces a critical property for producing reliable aggregate area statistics. Inter-fold variability in recall and precision remains moderate (Fold 3: recall 91.2%, precision 85.5%; Fold 4: recall 85.3%, precision 85.0%), consistent with the overall low dispersion reported in Table 7. We do not attempt to attribute these differences to specific spatial or spectral characteristics of the underlying folds, as this would require a dedicated analysis of fold composition that falls outside the scope of the present study.

Under the shared reference configuration, the three architectures achieve statistically equivalent performance (U-Net: 74.8±1.5 %, DeepLabV3 + : 75.0±1.6 %, SegFormer-B2: 75.3±2.3 % IoU; maximum spread < 0.5 pts, well below the significance threshold established by the ablation tests), confirming that architecture selection is secondary to training strategy design in this data regime. The choice of SegFormer-B2 as the operational architecture is therefore not grounded in a quantitatively superior cross-validation score, but rests on two complementary practical considerations documented in Section 3.2: (i) SegFormer-B2 produces spatially coherent predictions with systematically fewer false positives on bare mudflat substrates, a property that reduces commission bias in surface area estimation; and (ii) its lower sensitivity to the absence of augmentation, relative to CNN-based architectures, provides greater robustness across image acquisition conditions where photometric properties may vary between annual BD ORTHO^®^ cycles. Under strictly equivalent quantitative performance, these operational properties constitute the effective selection criterion.

The F1-score of 86.8% can be interpreted as a direct proxy for pixel-level agreement between model predictions and the reference labels. This implies that approximately 13% of pixel-level assignments are erroneous, distributed roughly equally between omission and commission. At the scale of the Bay, where *Spartina*-dominated communities have been estimated to cover 60–85% of intertidal vegetation surfaces [25], this translates into a surface area estimation uncertainty on the order of 10–15%. This level of uncertainty remains well within the range of inter-annual variability documented in diachronic photo-interpretation studies [29,31] and is substantially lower than the uncertainty inherent to field-based visual delineation in mixed *Spartina*, *Halimione*, *Limonium* assemblages, where even expert operators report significant inter observer disagreement.

The model was subsequently deployed in inference mode across the full intertidal extent of the Bay to produce a wall-to-wall *Spartina* prediction map from BD ORTHO^®^ CIR imagery and LiDAR HD DSM. The predicted surfaces were confronted with the existing labelled reference data assembled from drone photogrammetry, GNSS field surveys, and diachronic photo-interpretation. This comparison serves a dual purpose: validating the spatial coherence of the predictions beyond the cross-validation patches, and providing an independent estimate of the current *Spartina* extent that can be directly integrated into the PNRA management framework. For coastal managers, the operational implication should be stated with the appropriate nuance: an F1-score of 86.8% with balanced precision and recall indicates that, **for sites resembling those already mapped**, the predicted surface areas can be trusted as reliable estimates of colonised extent, updatable annually at near-zero marginal cost as new BD ORTHO^®^ acquisitions become available. As shown by the leave-one-site-out validation reported in Section 3.5, this level of accuracy should not be assumed to extrapolate unchanged to a genuinely new, previously unmapped site, where F1-scores ranging from 67.1% to 88.5% depending on the site (mean 77.8±6.9%) are a more realistic expectation until the site-specific model is retrained or fine-tuned on local labels. With this caveat, the pipeline nonetheless transforms *Spartina* monitoring from a labour intensive and time-consuming, episodic exercise into a reproducible, semi-automated workflow across already-characterised sites.

**Fig 9:**
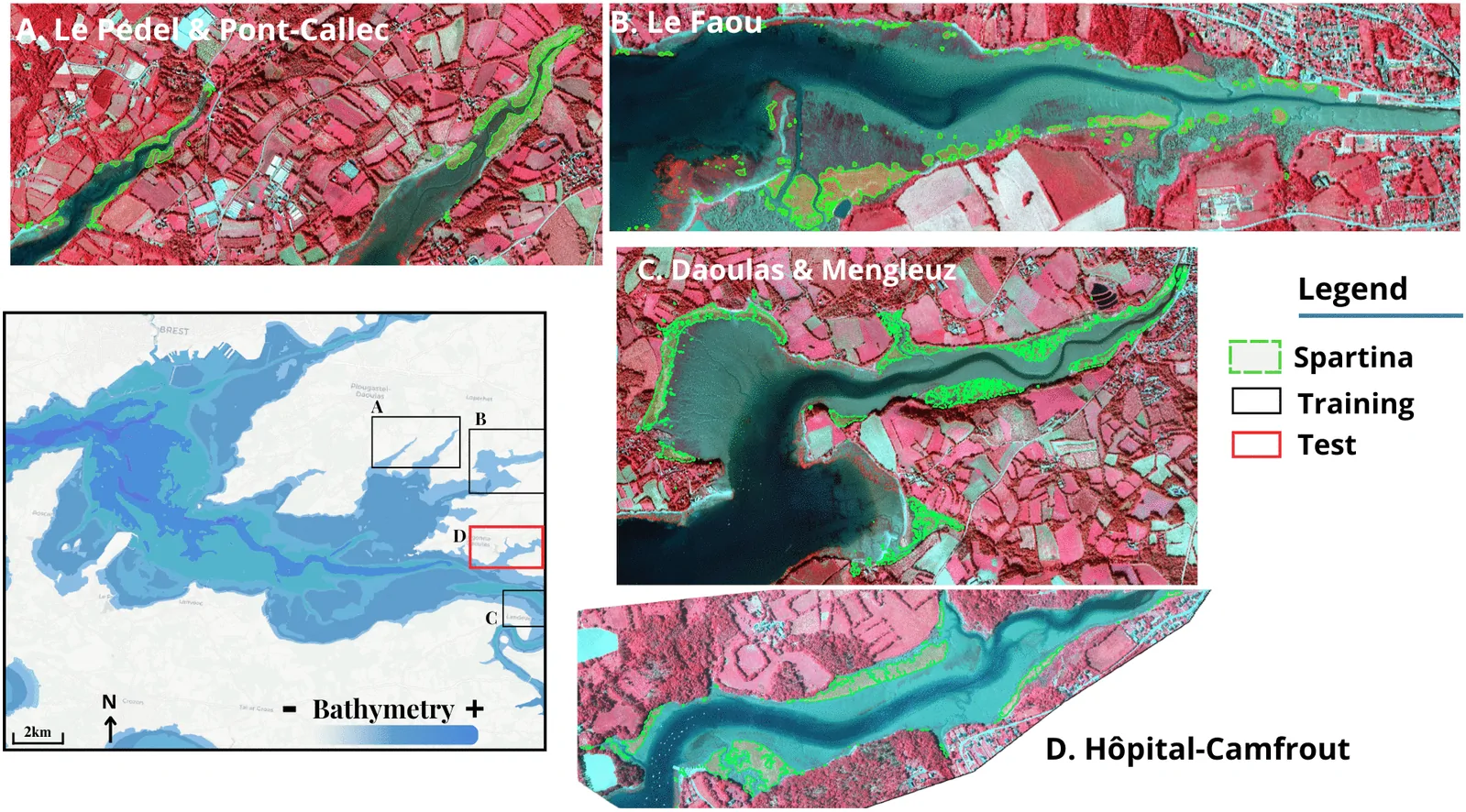
Spatial distribution of *Spartina* within the training and testing subsets in the Bay of Brest. Results were generated using the SegFormer-B2 architecture based on the optimal training strategy, which employed full encoder fine-tuning combined with Dice+BCE loss, cosine annealing, and basic data augmentation over 150 epochs. Basemap: IGN BD ORTHO^®^, Licence Ouverte / Etalab 2.0.

The wall-to-wall inference produced by the SegFormer-B2 model under the selected operational configuration was applied across all study sites in the BoB, covering both the training/validation zones (Le Pédel & Pont-Callec, Le Faou, Daoulas–Mengleuz) and an independent test site (Hôpital-Camfrout) for which no labelled reference data were used during model training (Fig 9). The resulting surface area estimates are summarised in Table 8.

**Table 8:** Summary of *Spartina* surface area estimates across study sites in the Bay of Brest.

Set	Site	Predicted (ha)	Reference (ha)	Δ (ha)	Lower (ha)	Upper (ha)
Train/Val	Le Pédel & Pont-Callec	8.3	7.95	+0.35	—	—
	Le Faou	10.8	10.20	+0.60	—	—
	Daoulas–Mengleuz	18.7	18.00	+0.70	—	—
Test	Hôpital-Camfrout	6.5	6.35^a^	+0.15	5.5	7.5
**Total**		**44.3**				

• Mean overestimation bias on train/val sites: +4.7%±1.0% (MAE = 0.55 ha).

^a^Reference surface at Hôpital-Camfrout derived from manual photo-interpretation on BD ORTHO ® CIR orthoimage; relative error = +2.4%. Uncertainty bounds correspond to ±15% of the predicted value (~85% confidence), accounting for classification accuracy and systematic bias.

On the three training/validation sites, where reference labels derived from drone photogrammetry and DGPS field surveys are available, the model predicts 8.3 ha, 10.8 ha, and 18.7 ha of *Spartina* cover for Le Pédel & Pont-Callec, Le Faou, and Daoulas–Mengleuz respectively, against reference values of 7.95 ha, 10.20 ha, and 18.00 ha (Table 8). The resulting overestimation bias is consistent across the three labelled sites (+4.4%, + 5.9%, and +3.9% respectively; mean +4.7%±1.0%, MAE = 0.55 ha), and aligns with the slightly higher recall than precision documented in the cross-validation results (Table 7). This systematic positive bias is operationally preferable to an underestimation bias for conservation management, as it reduces the risk of overlooking colonised areas. However, several caveats should be noted. First, the bias is characterised on only three sites, which precludes robust statistical generalisation; with such a small sample, the apparent stability of the overestimation rate (±1.0%) may partly reflect the limited morphological diversity sampled rather than a truly invariant model property. Second, re-predicting on training zones introduces an inherent optimistic evaluation bias, as the model has been exposed to spectral patterns from these sites during training although the consistency of the bias magnitude with the independent cross-validation metrics suggests that the model has learned transferable *Spartina* signatures rather than site-specific artefacts.

Moreover, the reference labels for several sites were produced from field campaigns conducted in 2025 or earlier [29,31], whereas the BD ORTHO^®^ imagery dates from 2024. The positive Δ values observed in Table 8 may therefore partly reflect actual *Spartina* expansion between the labelling date and the image acquisition date, rather than pure classification error. This temporal offset introduces an additional source of uncertainty in the per-site accuracy assessment: patches flagged as *Spartina* by the model but absent from the reference labels could correspond to genuine colonisation not yet captured by the labelling campaign, to commission errors, to residual registration error between the label and image sources, or to labelling error. Disentangling these explanations would require contemporaneous field validation, which was not available for all sites at the time of writing.

On the spatially withheld test site of Hôpital-Camfrout, the predicted surface of 6.5 ha can be compared to the 6.35 ha derived from manual photo-interpretation on BD ORTHO^®^ CIR orthoimage, yielding a relative error of +2.4%. This reference was derived from manual photo-interpretation on the same BD ORTHO^®^ CIR imagery used as model input, which introduces a partial circularity: both the prediction and the reference share the same spectral information base. The + 2.4% relative error therefore measures agreement between the model and a human interpreter working on identical imagery, rather than agreement with a fully independent ground truth. A rigorous independent validation would require contemporaneous GNSS or drone-based field delineation, which was not available for this site at the time of writing and constitutes a direction for future work. This near-neutral deviation provides empirical support for the ±15% uncertainty bounds estimated from pixel-level classification accuracy (F1 ≈85%) and systematic bias characterised on the labelled sites. It should nonetheless be acknowledged that the relationship between pixel-level F1-score and landscape-scale surface area error is not strictly linear: classification errors in remote sensing imagery tend to be spatially autocorrelated, concentrated along patch boundaries and in transitional halophyte assemblages, rather than independently distributed across pixels. Commission and omission errors therefore do not necessarily compensate at the landscape scale as they would under an i.i.d. assumption, and surface area estimates should be interpreted accordingly.

The total predicted *Spartina* cover across all monitored sites amounts to approximately 44.3 ha. Despite the methodological caveats outlined above, this figure provides local stakeholders and coastal managers, in particular the PNRA with a spatially explicit, reproducible, and internally consistent baseline of *Spartina* extent in the BoB, derived entirely from routinely updated aerial photography (BD ORTHO^®^) and nationally available LiDAR HD elevation data. The process can be fully automated and repeated at each new acquisition cycle, transforming *Spartina* monitoring from an episodic, labour-intensive exercise into a systematic, near-zero-cost operational workflow. For coastal managers, this means that surface area estimates comparable in magnitude to those obtained through multi-day DGPS surveys can now be produced in a matter of hours, enabling annual tracking of *Spartina* colonisation dynamics across the entire BoB with the explicit understanding that the reported areas carry an uncertainty envelope of approximately ±10−−15% that should be factored into management decisions.

The resulting wall-to-wall prediction map is publicly available as an interactive geovisualisation service, allowing readers to explore the 20 cm-resolution predictions across the entire BoB [42].

### 3.4. Contribution of spectral and elevation inputs (channel ablation)

The configurations reported above all use the full four-channel stack (R, G, NIR, DSM). To isolate the respective contributions of spectral and elevation information, and thereby to determine whether the observed performance derives from the segmentation model or simply from a strong ecological signal carried by elevation, we retrained the selected operational configuration under three input regimes that differ only in the channels supplied to the network: CIR-only (R, G, NIR), DSM-only (elevation alone), and the full CIR  +  DSM reference. All other factors were held constant (SegFormer-B2, Dice+BCE, dropout 0.2, cosine annealing, basic augmentation, 150 maximum epochs, patience 20, identical five-fold partition). Channel selection was applied before per-patch normalisation, so that the intensity statistics used for scaling are computed only over the channels actually supplied to the network, avoiding cross-contamination between regimes. Results are reported in Table 9.

**Table 9:** Channel ablation: contribution of spectral (CIR) and elevation (DSM) inputs. All configurations use SegFormer-B2 under the operational training strategy and the same five-fold partition; only the input channels differ. ΔIoU is expressed relative to the CIR  +  DSM reference.

Input channels	Ch.	IoU (%)	ΔIoU	F1 (%)	Prec. (%)	Rec. (%)	OA (%)
CIR + DSM (reference)	4	76.7 ± 2.1	—	86.8 ± 1.3	85.9 ± 1.2	87.7 ± 2.2	93.4 ± 0.5
CIR-only (R, G, NIR)	3	75.0 ± 2.8	−1.7	85.7 ± 1.8	84.7 ± 2.3	86.7 ± 1.6	92.9 ± 0.6
DSM-only (elevation)	1	54.2 ± 3.1	−22.5	70.2 ± 2.6	67.0 ± 5.7	74.4 ± 3.4	84.4 ± 1.5

Two results stand out. First, elevation alone is genuinely informative but clearly insufficient: DSM-only reaches 54.2±3.1% IoU, more than twice the value of 24.8% that a degenerate all-positive predictor would obtain at the observed class prevalence, confirming that the LiDAR-derived surface elevation carries real discriminative signal for *Spartina* rather than noise. It nevertheless falls 22.5 pts short of the full stack, and its precision is both the lowest and the most variable of the three regimes (67.0±5.7%), indicating substantial commission errors when elevation is the only available cue. Second, and more unexpectedly, removing the DSM entirely costs only 1.7 pts IoU (1.1 pts F1): the CIR-only model reaches 75.0±2.8% IoU, within the dispersion of the reference configuration. The answer to the question motivating this experiment is therefore that the reported performance is driven predominantly by the spectral channels, not by elevation: the near-infrared band, which strongly separates dense vegetation from bare intertidal substrate, accounts for most of the discriminative power, and the marginal contribution of the DSM in the present setup is small.

We emphasise that this small marginal contribution should not be read as evidence that elevation is ecologically uninformative for *Spartina* mapping, since the DSM-only result demonstrates the contrary. The interpretation of this apparent discrepancy, and in particular the role of the input-level fusion strategy adopted here, is developed in Section 4.4.

### 3.5. Leave-one-site-out validation of the operational configuration

The five-fold cross-validation results reported above rely on a random, patch-level partitioning of the dataset: although patches are strictly non-overlapping, patches from the same site can appear in both the training and validation subsets of a given fold (Section 4.5). To directly test whether this design overestimates the model’s ability to generalise to a genuinely new, previously unmapped site, we conducted a leave-one-site-out (LOSO) validation of the selected operational configuration (SegFormer-B2, Dice+BCE, dropout 0.2, cosine annealing, basic augmentation, 150 maximum epochs, patience 20). Each of the five labelled sites (Daoulas–Mengleuz, Le Faou, Keroullé, Le Pédel, Pont-Callec) was in turn held out in its entirety as the validation set, while the model was trained from scratch on the patches from the four remaining sites. Site membership was determined for each patch from its own georeferenced extent (rather than from filename conventions, which do not encode site identity in this dataset) via a spatial join against the site boundary polygons used for patch extraction.

Table 10 reports the per-site results. Averaged across the five held-out sites, the model achieved a mean IoU of 64.2±9.3% (95% CI: [51.2, 77.1]) and a mean F1-score of 77.8±6.9% (95% CI: [68.2, 87.3]), against 76.7±2.1% IoU and 86.8±1.3% F1 under the random five-fold protocol reported in Table 7 (a drop of 12.5 and 9.0 pts respectively), and a standard deviation across sites approximately 4.4 times larger for IoU. Performance also varied substantially between held-out sites, from 50.5% IoU (Pont-Callec) to 79.5% IoU (Le Pédel), a 28.9-point range that random cross-validation cannot expose by construction, since every random fold contains a representative mixture of patches from all sites. The background class remained comparatively stable across held-out sites (mean IoU 84.6±5.1%), while the target *Spartina* class carried essentially all of the observed variability (mean IoU 64.2±9.3%, identical to the headline value since this is a binary segmentation task), indicating that the generalisation gap is specific to recognising *Spartina* in an unfamiliar site rather than a general degradation of segmentation quality. Precision and recall were also unevenly affected: recall exceeded precision by 4.8 to 14.6 pts on four of the five held-out sites (Daoulas–Mengleuz, Le Faou, Keroullé, Pont-Callec), indicating a tendency towards over-prediction (commission errors) when the model is applied to a site absent from training; Le Pédel was the exception, with a near-balanced, slightly precision-biased profile (precision 89.5%, recall 87.6%) comparable to the random cross-validation regime.

**Table 10:** Leave-one-site-out (LOSO) validation of the operational configuration (SegFormer-B2). Each row corresponds to one site held out in its entirety as validation, with the model trained on the four remaining sites. $N_{train}$ and $N_{val}$: number of patches. Epochs: training epochs completed before early stopping (patience 20, maximum 150).

Held-out site	$N_{train}$	$N_{val}$	IoU (%)	F1 (%)	Prec. (%)	Rec. (%)	Epochs
Daoulas–Mengleuz	484	326	62.0	76.5	69.9	84.5	47
Le Faou	527	283	62.4	76.9	73.7	80.3	41
Keroullé	752	58	66.4	79.8	76.2	83.9	51
Le Pédel	732	78	79.5	88.5	89.5	87.6	29
Pont-Callec	745	65	50.5	67.1	64.8	69.6	31
**Mean ± Std**			64.2 ± 9.3	77.8 ± 6.9	74.8 ± 8.3	81.2 ± 6.2	40 ± 9

We do not have direct evidence to attribute the particularly low generalisation performance on Pont-Callec to a specific cause, and we explicitly refrain from speculating beyond what the data support. We note, without drawing a firm conclusion, that Pont-Callec and Le Pédel share the same ground-truth labelling source (digitised from IGN BD ORTHO^®^ orthophotographs without drone support; Table 1) yet occupy opposite extremes of the LOSO performance range, which argues against labelling source alone as the explanation. Site-specific ecological factors (e.g., a distinct mixture with *Halimione portulacoides* or *Limonium* spp., a different colonisation front morphology, or a different tidal exposure at the time of BD ORTHO^®^ acquisition) are plausible contributors, but confirming any of them would require a dedicated site-by-site investigation that falls outside the scope of the present study. This pattern is discussed further in Section 4.5.

## 4. Discussion

### 4.1. Training strategy dominance and architectural convergence

The central finding of this study challenges the prevailing focus on architectural novelty in the remote sensing literature, where models are typically compared under a single fixed protocol [21–23,43]. Under suboptimal strategies, all three architectures converge to similarly degraded performance. This result is consistent with Safonova et al. [44], who identified training strategy design as the primary lever for small-data remote sensing, and with Ma et al. [24], who showed that loss function choice alone can induce 5–7% Dice variations across architectures.

The convergence of architectures from three structurally distinct families is itself informative. The theoretical advantage of Transformer-based architectures lies in their capacity to model long-range spatial dependencies through global self-attention [18], a property that yields substantial gains on large-scale benchmarks where contextual information spans hundreds of pixels. On a training set of 810 patches at 20 cm GSD with patch extents of 44.8 m × 44.8 m, this capacity is unlikely to be fully expressed: the limited data volume restricts the diversity of spatial configurations available to the model, and the contextual window is sufficient for individual colonisation fronts but insufficient for the landscape-level patterns that Transformers exploit at larger extents. Under these conditions, training strategy, which governs convergence dynamics, regularisation, and gradient flow, becomes the dominant determinant of whether any architecture reaches its performance ceiling. Our IoU values (74–76%) are broadly consistent with VHR coastal vegetation mapping studies [45]; higher values reported by Tan et al. [46] for the Yellow River Delta relied on multi-temporal Sentinel-2 and substantially larger datasets, a fundamentally different operational context. For practitioners: on small geospatial datasets, investing effort in training strategy design yields greater returns than selecting a more complex architecture [47–49].

### 4.2. Architecture-specific training sensitivities: encoder freezing and implicit regularisation

Two ablation axes revealed pronounced architecture-dependent responses that carry direct practical implications.

The Freeze  +  Warmup strategy produced the largest single effect in the ablation, confirming that encoder freezing is actively harmful when the domain shift between source (ImageNet RGB) and target (CIR  +  DSM) is substantial. Frozen early-layer filters cannot adapt to near-infrared reflectance and LiDAR elevation features absent from the pre-training distribution. This aligns with Neumann et al. [50] and Wurm et al. [51], who demonstrated that full fine-tuning is indispensable in domain-shifted remote sensing contexts. The smaller degradation for SegFormer likely reflects the greater flexibility of its mixed depthwise convolution-based encoder compared to fixed early CNN filters. The common heuristic of freezing encoders to prevent overfitting on small datasets [52] should be applied with extreme caution in multi-modal or non-RGB remote sensing contexts.

The regularisation axis revealed a complementary asymmetry: SegFormer collapsed at dropout  = 0.5 while CNN architectures remained unaffected, yet SegFormer was uniquely robust in the absence of augmentation. Both observations are consistent with the implicit regularisation properties of the MiT-B2 encoder, which integrates attention dropout and stochastic depth internally [18]; adding external dropout produces over-regularisation, while self-attention implicitly diversifies feature representations, compensating for limited input diversity [53,54]. For practitioners: Transformer-based models on small geospatial datasets should use minimal external regularisation (dropout ≤0.2) and can achieve robust performance with basic augmentation alone.

### 4.3. The Tversky loss paradox

The Tversky loss (α=0.3, β=0.7) degraded all three architectures by ~4.5 pts IoU, despite being widely recommended for imbalanced segmentation [55]. This result is explained by the moderate class imbalance in our dataset (~24.8% *Spartina* pixels): the Tversky loss was designed for extreme imbalance where the target class occupies fewer than 5% of pixels [55,56]. Yeung et al. [57] confirmed that Focal Tversky does not consistently improve performance under moderate imbalance, and Ma et al. [24] showed that compound losses (Dice+BCE, DiceFocal) provide the most robust performance across varying imbalance ratios. The α/β hyperparameters were set to the values most commonly reported in the segmentation literature (α=0.3, β=0.7), which correspond to the default implementation in major frameworks such as MMSegmentation [58], and are recommended by Yeung et al. [57] specifically to up-weight false negatives and improve recall under severe class imbalance. These values were not further optimised via grid search in the present study. However, the degradation magnitude observed here is inconsistent with a mere calibration artefact: it reflects a structural mismatch between the asymmetric penalty designed for extreme imbalance regimes (foreground  <  5% of pixels, as in lesion or vessel segmentation) and the moderate imbalance of the present dataset. Under moderate imbalance, the strong false-negative penalisation induced by β=0.7 produces systematic over-segmentation that degrades IoU without improving the precision–recall trade-off relevant to surface area estimation. We acknowledge that testing a single parameter combination (α=0.3, β=0.7) does not characterise the full Tversky family. However, these are the most widely reported default values [57,58], and the magnitude of the degradation (~4.5 pts) under a moderate 24.8% class imbalance suggests a structural mismatch rather than a tuning artefact. A systematic grid search over (α, β) pairs is reported as a direction for future work.

### 4.4. Interpreting the modest marginal contribution of elevation: An input-level fusion limitation

The channel ablation (Section 3.4) produced an apparent tension that deserves careful interpretation. Elevation alone supports a non-trivial segmentation of *Spartina* (54.2±3.1% IoU, more than twice the degenerate baseline), yet adding it to the spectral channels improves performance by only 1.7 pts IoU. A first reading would conclude that the DSM is largely redundant with the spectral signal in this intertidal context. We consider a second, non-exclusive explanation at least as plausible: that the marginal gain is limited less by the intrinsic information content of the DSM than by the difficulty of integrating it, at the input level, into a network whose representations were pre-trained on three-channel photographic imagery.

Three arguments support this reading. First, the fusion strategy adopted here is early (input-level) fusion, in which the DSM is concatenated to the spectral bands as an additional channel. This is the simplest multi-modal strategy, but it constrains the network to learn a joint representation from the very first layer and does not allow it to exploit cross-modal complementarity at deeper levels; early fusion is moreover generally described as best suited to modalities that share semantic similarities, which reflectance and elevation only partially do [59]. Second, early fusion interacts poorly with transfer learning: a network whose input layer is modified to accept a supplementary channel cannot fully inherit the pre-trained weights of that layer, since the pre-training distribution contains no analogue of an elevation band [60]. In the present implementation the DSM channel is initialised by replicating the weights of a spectral channel, a pragmatic but semantically arbitrary choice that the fine-tuning stage must subsequently undo. This penalty applies specifically to the CIR  +  DSM configuration and not to CIR-only, which inherits its input-layer weights unchanged, so the two regimes do not start from equivalent optimisation conditions. Third, the benchmark results of Xiong et al. [61] on RGB-height segmentation are consistent with this interpretation: in their Transformer-based experiments, naive early fusion improves mIoU over the RGB-only baseline by approximately 5.6 pts, whereas feature-level, late, and intermediary fusion strategies extract systematically more value from the same elevation modality. Their analysis also reports that spectral input generally outperforms height input when each is used alone, which matches the ordering observed here.

We therefore interpret the modest +1.7 pts contribution of the DSM as a lower bound on the value of elevation information for *Spartina* mapping under the present architecture and fusion strategy, rather than as a measure of its ecological relevance. We note that we cannot disentangle the respective weights of genuine spectral-elevation redundancy and of fusion inefficiency with the experiments reported here: doing so would require comparing input-level fusion against feature-level or late fusion designs under otherwise identical conditions, which we did not conduct.

This limitation points to a concrete direction for future work. Dual-branch architectures that encode spectral and elevation modalities separately before fusing them at the feature or decision level, cross-modal attention mechanisms that allow each modality to modulate the other, and modality-specific normalisation schemes better adapted to the physical nature of elevation data than the percentile scaling used here, all constitute plausible routes to exploiting the DSM more effectively. Given that elevation alone already supports a 54% IoU segmentation, and given the strong ecological rationale linking *Spartina* distribution to the tidal inundation window, we consider it likely that a better-designed fusion strategy would recover a larger share of that information than the 1.7 pts obtained here. Testing this hypothesis is a priority for subsequent work, and would also clarify whether elevation contributes disproportionately in precisely the transitional zones where the present model is weakest (Section 3.2).

### 4.5. Spatial autocorrelation and validation strategy

Recent work has demonstrated that spatially autocorrelated training and validation samples can substantially inflate performance metrics in convolutional neural network-based classification and segmentation tasks [36,37]. When nearby patches share similar spectral signatures, texture, and landscape context, a model may appear to generalise well in cross-validation while in reality exploiting spatial similarity rather than learning transferable class-discriminative features. This concern is particularly relevant in remote sensing, where environmental gradients produce strong spatial structure in the input data [62]. This issue has been shown to inflate metrics by up to 28% in tree species segmentation from drone imagery [36], and to produce entirely misleading predictive skill assessments in large-scale biomass mapping [63].

The absence of adjustment for multiple comparisons in the original analysis was addressed by applying a Benjamini–Hochberg correction across all twelve Friedman tests; no previously reported significant effect became non-significant after adjustment, confirming the robustness of the main conclusions to this correction.

To directly test this concern rather than relying on qualitative mitigating arguments, we conducted a leave-one-site-out (LOSO) validation of the selected operational configuration, reported in full in Section 3.5 and Table 10. Each of the five labelled sites was held out in its entirety as validation, with the model trained on the remaining four. The result confirms the reviewer’s concern directly: mean performance dropped from 76.7±2.1% IoU under random five-fold cross-validation to 64.2±9.3% IoU under LOSO (a −12.5 pts shift), and the standard deviation across folds increased approximately 4.4-fold. This indicates that the non-overlapping patch-level partitioning used throughout the ablation study, while eliminating pixel-level leakage, does not eliminate site-level autocorrelation: patches drawn from the same estuary at the same acquisition date share illumination conditions, tidal state, sediment colour, and a site-specific mixture of halophytic species, all of which the model can partially exploit when a patch from the same site is available during training. The random five-fold estimates reported elsewhere in this article (Tables 6 and 7) should accordingly be read as an optimistic, same-distribution estimate, representative of deployment on sites that resemble those already mapped, rather than as an estimate of extrapolation to an entirely new, previously unmapped site. The LOSO results provide the latter, stricter estimate, and should be preferred whenever the intended application involves site-level extrapolation.

LOSO also exposed a degree of inter-site heterogeneity that random cross-validation is structurally unable to detect, since every random fold contains a representative mixture of patches from all sites by construction: held-out performance ranged from 50.5% IoU (Pont-Callec) to 79.5% IoU (Le Pédel), and this variability is concentrated almost entirely in the *Spartina* class (background IoU: 84.6±5.1% across held-out sites, versus 64.2±9.3% for *Spartina*). As detailed in Section 3.5, we did not find an explanatory factor that is fully consistent with this pattern: Pont-Callec and Le Pédel share the same ground-truth labelling source, yet occupy opposite extremes of the LOSO performance range. We therefore report this heterogeneity as an open empirical finding rather than attribute it to a specific mechanism, and identify a dedicated, site-by-site investigation of its determinants as a priority for future work.

An important limitation of the present LOSO experiment is that it was conducted on a single configuration, the selected operational setup, rather than repeated across the full ablation matrix (12×3 configurations). The argument advanced in this study, that the relative ranking of training strategies is unlikely to be substantially affected by autocorrelation bias because that bias would apply approximately equally across configurations sharing the same cross-validation protocol [64], is therefore not directly tested by the present LOSO results: site identity, the source of variability quantified here, is orthogonal to the training-strategy axes varied in the ablation, and repeating the full ablation under LOSO would be required to confirm that ranking-preservation assumption empirically. We identify this as the most direct follow-up to the present study.

Beyond the LOSO experiment, several other design choices constrain the practical impact of spatial autocorrelation on the conclusions drawn here. The multi-site sampling strategy distributes training data across five geographically distinct estuarine zones separated by several kilometres of rocky coastline, each with its own tidal regime, sediment characteristics, and halophytic assemblages, introducing substantial spectral and contextual diversity within each random fold. The non-overlapping patch extraction ensures pixel-level independence between train and validation subsets within a given fold, and the operational generalisation assessment (Section 3.3) additionally includes one entirely independent test site (Hôpital-Camfrout) that was never used during training or validation of any configuration, providing a further, if partial, check on transferability beyond the cross-validation patches.

### 4.6. Ecological constraints, transferability, and study limitations

**Ecological basis of dataset scale.** The restriction to a single study site reflects the ecological and geographical constraints inherent to the target object rather than a methodological choice. Zhou et al. [65] demonstrated that national-scale sub-metre mapping of *Spartina* is achievable along the Chinese coastline, where the species dominates thousands of hectares of continuous tidal flats. This approach is not transferable to the BoB context: *Spartina* colonisation is spatially fragmented across small estuarine inlets, each hosting distinct halophytic assemblages shaped by local tidal regime and competition dynamics [66]. The total colonised area (~44 ha) represents a fraction of the spatial extent available in Chinese or North American systems, and the mono-site design provides the controlled input conditions required to isolate training strategy effects from architectural design.

**Operational generalisation.** These estimates were produced at near-zero marginal cost from routinely maintained institutional imagery, demonstrating the operational readiness of the pipeline for annual monitoring cycles within the Bay of Brest. It should be noted, however, that the model presented here was trained exclusively on BoB conditions, including its specific tidal regime, sediment characteristics, halophytic assemblages, and spectral signatures. Direct application to other Atlantic estuaries without retraining would therefore not be appropriate. The methodology and training strategy recommendations derived from this study are nonetheless transferable: the nationwide availability of BD ORTHO^®^ and LiDAR HD provides the data infrastructure required to replicate this workflow in other fragmented coastal systems (Baie de Saint-Brieuc, Golfe du Morbihan, Bassin d’Arcachon), provided that site-specific labelled datasets are assembled and the full training pipeline is applied locally.

**Sensitivity to patch geometry.** All experiments used a fixed patch size of 224×224 pixels (44.8 m × 44.8 m). Larger patches (e.g., 512×512) could provide SegFormer access to broader landscape patterns currently truncated at patch boundaries [67], but would simultaneously reduce the number of available training samples. This trade-off was not explored here and constitutes a natural sensitivity analysis for future work [68].

**Remaining limitations.** The OFAT ablation design does not capture factor interactions; a full factorial or Bayesian optimisation approach [69] could reveal synergies missed here at substantially higher computational cost. Foundation model comparisons (SAM [70]; Prithvi [71],) and multi-class segmentation (*Spartina*/*Halimione*/*Limonium*) are natural follow-ups. The ±15% surface area uncertainty does not account for spatial autocorrelation of classification errors [72]; geostatistical error modelling would provide more rigorous confidence bounds.

## 5. Conclusion

This study set out to determine whether, under the realistic data constraints faced by coastal managers, the choice of segmentation architecture or the choice of training strategy has a greater impact on the automated detection of *Spartina* from routinely available aerial imagery. Through a systematic ablation of 180 training runs across three architectural families (U-Net, DeepLabV3 + , SegFormer-B2) and twelve training configurations evaluated by five-fold cross-validation, the answer is unambiguous: training strategy dominates. The performance gap between the best and worst strategy (~10 pts IoU) exceeds the inter-architecture spread (<1 pt) by an order of magnitude, demonstrating that the common practice of benchmarking architectures under a single fixed protocol can lead to misleading conclusions when datasets are small and domain-specific.

Two training decisions proved systematically detrimental regardless of architecture: encoder freezing, which produced catastrophic drops of −6 to −10 pts IoU due to the substantial domain shift between ImageNet RGB and the CIR  +  DSM input space; and the Tversky loss (α=0.3, β=0.7), whose asymmetric weighting degraded all models by ~4.5 pts under the moderate class imbalance. Conversely, standard composite losses and full end-to-end fine-tuning with cosine annealing provided robust, architecture-agnostic baselines. The regularisation axis further revealed that CNN and Transformer architectures respond to training decisions in fundamentally different ways: SegFormer-B2 collapsed under high external dropout yet tolerated the absence of augmentation, while the CNN-based models exhibited the opposite pattern a finding with direct implications for practitioners configuring training pipelines on small geospatial datasets.

Beyond these methodological insights, the operational dimension of this work is central. The selected configuration (SegFormer-B2, basic augmentation, Dice+BCE, full fine-tuning) achieved an F1-score of 86.8±1.3% with near-symmetric precision–recall balance, enabling wall-to-wall *Spartina* surface estimation across the BoB from BD ORTHO^®^ and LiDAR HD data alone. The characterised systematic bias of +4.7% on labelled training sites falls well within the variability inherent to diachronic photo-interpretation and field-based visual delineation. On the spatially withheld site of Hôpital-Camfrout, the model output agrees with manual photo-interpretation of the same imagery to within +2.4%; as this reference shares the same spectral input as the model, a fully independent field-based assessment is required to confirm operational accuracy and is planned as a priority next step. Within these bounds, the pipeline reduces the logistical overhead of *Spartina* monitoring considerably: surface area estimates previously requiring multi-day GNSS campaigns can now be produced in hours and updated at each new imagery cycle, transforming monitoring into a systematic, reproducible, and low-cost workflow for the PNRA and associated conservation stakeholders.

This operational capacity is all the more valuable given the intrinsic difficulty of *Spartina* as a geographical object. Unlike spectrally homogeneous land cover classes, *Spartina* in the BoB forms narrow, fragmented colonisation fronts at the low-schorre margin, interdigitating with spectrally similar halophytes *Halimione portulacoides*, *Limonium* spp. in transitional assemblages where even expert photo-interpreters struggle to delineate boundaries with confidence. Understanding *Spartina* dynamics is inseparable from understanding the coastal system as a whole: its colonisation patterns reflect tidal regime, sediment supply, estuarine morphology, and the competitive interactions with native vegetation that collectively shape the ecological trajectory of intertidal habitats. Automating its detection is therefore not merely a technical exercise in image classification, but a prerequisite for reading the ongoing transformation of these environments at the spatial and temporal scales required for effective management.

As coastal ecosystems face accelerating pressures from biological invasion, sea-level rise, and shifting sediment dynamics, the capacity to track vegetation changes across entire embayments rather than on isolated monitoring plots will become increasingly critical. The pipeline developed here offers a scalable framework that can be extended to other cartographically elusive intertidal objects (*Limonium humile* stations, seagrass meadows, intertidal biofilm communities) and transferred to other Atlantic estuarine systems where BD ORTHO^®^ and LiDAR HD coverage is nationally available. By demonstrating that rigorous training strategy design matters more than architectural sophistication, and by delivering an operational tool anchored in routinely maintained institutional data, this study bridges the gap between deep learning methodological research and the practical needs of coastal managers tasked with monitoring and adapting to the dynamics of invaded littoral systems.
