# Supplementary material for: Training strategy over architecture: A systematic ablation study for Spartina detection from aerial imagery with small geospatial datasets
Source: PLoS One. 2026 Sep 18;21(9):e0358464. doi: 10.1371/journal.pone.0358464 (PMC13588420; doi:10.1371/journal.pone.0358464)
Supplement: Supplementary file 1 [file pone.0358464.s001.pdf]

Response to Reviewers

Manuscript: PONE-D-26-28775 **Title:** Training strategy over architecture: A systematic ablation study for *Spartina* detection from aerial imagery with small geospatial datasets

---

Dear Dr. Hewitt, dear Reviewer,

Thank you for the careful reading of our manuscript and for the constructive comments. We are particularly grateful to Reviewer 1, whose two major points led us to run additional experiments that materially changed how we interpret and present our results. In both cases the new experiments confirmed the reviewer's concern rather than dismissing it, and we have revised the manuscript accordingly, including the abstract.

Below we respond to each point in turn. Reviewer comments are given in *italics*, our responses in plain text, with the corresponding manuscript changes indicated. Table and figure numbers refer to the final revised manuscript.

Reviewer 1 — Major concerns

1. Individual contribution of spectral and elevation information

The manuscript uses R, G, NIR, and DSM inputs, but does not demonstrate the individual contribution of spectral information and elevation information. A key missing experiment is comparison among CIR-only, DSM-only, CIR+DSM, and potentially classical machine-learning baselines. Without this, it remains unclear whether the observed performance is due to deep learning architecture or simply the strong ecological signal provided by elevation.

We agree that this experiment was missing, and we have now run it. We retrained the selected operational configuration under three input regimes that differ only in the channels supplied to the network, holding everything else constant (same architecture, same training strategy, same five-fold partition, channel selection applied before per-patch normalisation to avoid cross-contamination between regimes).

Input	IoU (%)	F1 (%)
CIR + DSM (reference)	76.7 ± 2.1	86.8 ± 1.3
CIR-only (R, G, NIR)	75.0 ± 2.8	85.7 ± 1.8
DSM-only (elevation)	54.2 ± 3.1	70.2 ± 2.6

The answer to the reviewer's question is that the reported performance is driven predominantly by the **spectral** channels, not by elevation: removing the DSM entirely

costs only 1.7 pts IoU. This is a correction to the framing of our original manuscript, which presented surface elevation as "a strong discriminant"; that claim is not supported by the data and has been rewritten as an expectation to be tested rather than an established fact.

We were careful not to over-correct in the other direction. Elevation alone reaches 54.2% IoU, more than twice what a degenerate all-positive predictor would obtain at our class prevalence (24.8%), so the DSM does carry real discriminative signal; its precision is also the lowest and most variable of the three regimes, indicating it produces meaningfully more commission errors when used alone. We discuss this apparent tension explicitly in a new Discussion subsection, and propose that the small marginal contribution of the DSM in the combined configuration reflects, at least in part, the limitations of input-level (early) fusion of a physically heterogeneous modality into an encoder pre-trained on three-channel imagery: the modified input layer cannot fully inherit the pre-trained weights, and the DSM channel must be initialised by an arbitrary heuristic (replicating a spectral channel's weights) that fine-tuning must subsequently undo, whereas the CIR-only model inherits its weights unmodified. We support this reading with three published sources on multi-modal fusion in segmentation (El Madawi et al. 2019; Xiong et al. 2023; Hurtado & Valada 2022), and state explicitly that we cannot separate genuine spectral–elevation redundancy from fusion inefficiency with the experiments reported here, identifying improved fusion strategies as a priority for future work.

Changes: New Results subsection "Contribution of spectral and elevation inputs (channel ablation)" with Table 9; new Discussion subsection "Interpreting the modest marginal contribution of elevation: an input-level fusion limitation"; the Methods description of the DSM rewritten to remove the unsupported "strong discriminant" claim; three new references added; the abstract now reports the channel ablation.

---

2. Spatial cross-validation and transferability

I am a bit concerned about the spatial cross-validation analysis. The current validation strategy may still overestimate model transferability because training and validation patches are spatially close and originate from the same sites. [...] Please consider adding leave-one-site-out validation, spatial block cross-validation, or distance-buffered validation to better assess performance under realistic deployment conditions.

The reviewer is right, and our original response to this concern (a set of qualitative mitigating arguments explaining why leave-one-site-out had not been adopted) was not an adequate substitute for the experiment itself. We have now run the leave-one-site-out (LOSO) validation.

Each of the labelled sites was withheld in its entirety as validation, with the model retrained from scratch on the remaining sites. Because our patch filenames are purely sequential and carry no site information, site membership was determined from the georeferenced extent of each patch via a spatial join against the site boundary polygons; all 810 patches were successfully assigned.

The result confirms the concern:

- Mean IoU drops from 76.7 ± 2.1% (random five-fold) to **64.2 ± 9.3%** (LOSO), a −12.5 pts shift, with the standard deviation across folds increasing roughly **4.4-fold**.
- Per-site performance ranges from **50.5% IoU (Pont-Callec) to 79.5% IoU (Le Pédel)**, a 28.9-point spread that random cross-validation cannot expose by construction.
- The variability is concentrated in the *Spartina* class; the background class remains comparatively stable (84.6 ± 5.1%).

We now present the random five-fold estimates as an optimistic, same-distribution estimate appropriate for sites resembling those already mapped, and the LOSO estimates as the stricter estimate to be preferred whenever site-level extrapolation is intended. The abstract, the operational implications paragraph, and the Discussion have all been revised accordingly; in particular, the sentence stating that the predicted surfaces "can be trusted as reliable estimates" has been qualified to specify sites resembling those already mapped.

We also report honestly what we could not explain. Pont-Callec generalises poorly, but we found no factor fully consistent with the pattern: Pont-Callec and Le Pédel share the same ground-truth labelling source (photo-interpretation only, no drone) yet sit at opposite extremes of the LOSO range. We report this as an open empirical finding rather than attribute it to a mechanism we cannot demonstrate.

One limitation is stated explicitly: the LOSO experiment was run on the selected operational configuration only, not repeated across the full ablation matrix. Our argument that the *relative ranking* of training strategies is robust to autocorrelation bias is therefore not directly tested by these results, and we now say so rather than leaving it implicit.

Changes: New Results subsection "Leave-one-site-out validation of the operational configuration" with Table 10; Discussion subsection on spatial autocorrelation substantially rewritten (the previous justification for not performing LOSO has been replaced by the results and their interpretation); abstract and operational implications revised.

Reviewer 1 — Minor concerns

3. Taxonomic terminology

The manuscript alternates among Spartina, Spartina alterniflora, Sporobolus and Sporobolus alterniflorus. Introduce the accepted name and synonym once, and then use one name consistently.

Corrected. The accepted name and its synonym are introduced once, at first mention in the Introduction (*Sporobolus alterniflorus*, syn. *Spartina alterniflora*), and "*Spartina*" is used consistently everywhere else, including the abstract, figure captions, and conclusion.

---

4. "Obione" vs *Halimione portulacoides*; "endemic"

"Obione" is used while later sections use Halimione portulacoides. Use consistent taxonomic terminology. Also reconsider describing all the listed plants or communities as "endemic."

Both corrected. "Obione" has been replaced by *Halimione portulacoides* throughout. The "endemic" qualifier has been removed from the list of co-occurring halophytes, where it was not justified; it is retained only for *Limonium humile*, where it is accurate.

---

5. Inconsistent list of study sites

The study-site description lists Daoulas, Mengleuz/Rosméléc and Le Faou, whereas Figure 2 and the dataset section also include Le Pédel, Pont-Callec and Hôpital-Camfrout. Describe all training, validation and test sites consistently.

This was a real inconsistency and we have rewritten the study-site section to resolve it. All six labelled zones are now listed and described explicitly: Daoulas, Mengleuz/Rosmélec, Le Faou, Keroullé, Le Pédel, and Pont-Callec. Hôpital-Camfrout is described separately as the independent test site, since it contributes no labelled training patches.

We also identified and documented a source of ambiguity that had contributed to the confusion, including in our own reporting: Mengleuz/Rosmélec is a distinct inlet but lies within the broader Daoulas estuarine system, and is therefore sometimes reported as part of Daoulas. This is now stated explicitly at the start of the study-site section.

While preparing the leave-one-site-out analysis we further found that the "Le Faou" column of the fold composition table (Table 2) aggregated Le Faou (283 patches) and the adjacent Keroullé inlet (58 patches). We verified this arithmetically against the georeferenced site assignment: all other columns match exactly under both

assignments. Rather than silently altering the table, we documented this aggregation in a table footnote and report the two zones separately in the LOSO analysis (Table 10), where site membership was reassigned from the georeferenced extent of each patch.

---

6. Digitisation scale of 1:144

A digitisation scale of 1:144. Please verify the value and explain how digitisation consistency was assessed.

We have verified the value: 1:144 is correct and intentional. It was adopted to match the scale used by Le Meur (2024) for the same estuarine sites, so that the resulting layers remain directly comparable with that earlier mapping. We have added an explanation of how consistency was ensured: digitisation was performed at this fixed reference scale rather than at a variable, screen-zoom-dependent scale, following the same protocol as the earlier study.

---

7. nDSM vs DSM

The input is first called a "normalised digital surface model," but it is subsequently described as absolute surface elevation. An nDSM and a DSM are different products. Use the correct term throughout.

Corrected. The product used is an absolute DSM, not a normalised nDSM. The residual occurrence of "normalised" has been removed; the term "DSM" is now used consistently.

---

8. Extrapolation to Pléiades, SPOT-7, Sentinel-2

The claim that a model trained on 20-cm aerial CIR imagery can be directly extrapolated to Pleiades, SPOT-7 or Sentinel-2 is too strong. [...] Present this as a potential future transfer experiment requiring retraining and validation.

Agreed and rewritten. The passage no longer claims direct extrapolation. It now states that these sensors differ substantially in spatial resolution, spectral response, and acquisition geometry, that transfer would require dedicated retraining and independent validation on sensor-specific data, and that we identify this as a direction for future work rather than a capability demonstrated here.

---

9. LiDAR acquisition dates and effective resolution

State the LiDAR acquisition dates. Resampling a 50-cm DSM to 20 cm does not produce an effective spatial resolution of 20 cm, so this distinction should be made explicit.

The acquisition campaign has been added: IGN LiDAR HD national programme, Brittany acquisition campaign, winter 2024–2025. We note that IGN publishes day-level acquisition dates only per individual 1 km tile, not per region; we report the campaign period, which is the level at which the information is authoritatively available for the study area.

On the resampling point, the reviewer is correct and the manuscript already distinguished the native 50 cm resolution from the 20 cm resampled grid; we have kept this distinction explicit, stating that bilinear interpolation to 20 cm GSD was performed to match the BD ORTHO® grid for stacking purposes and does not create additional elevation detail.

---

10. Class imbalance: 30% vs 24.8%

The dataset is described as containing approximately 30% Spartina pixels, whereas the fold analysis reports approximately 24.8%. Explain whether different spatial masks, subsets or denominators were used.

There was no methodological difference; the "≈30%" figure was an imprecise approximation carried over from an earlier draft. The measured value is 24.8%, as reported in the fold composition table. We have harmonised all occurrences in the manuscript to 24.8%.

---

11. Missing training details in Methods

Essential training details are missing, including maximum epochs, early-stopping patience etc. Early stopping and a maximum of 375 cumulative epochs appear only later in the Results.

Added. The Methods now state, for the ablation study, a maximum of 75 training epochs with an early-stopping patience of 20 epochs (monitored on validation IoU), both in the text and in the Table 3 caption. We also clarified that the "375 epochs" figure reported in the Results is a *cumulative* total across the five folds (5 × 75 maximum), not a per-fold maximum, which was ambiguous in the original text.

We have additionally added a dedicated "Statistical analysis" subsection to the Methods, reporting the exact software used (Python 3.12.13, NumPy 2.4.4, pandas 3.0.3, SciPy 1.18.0, scikit-learn 1.9.0, PyTorch 2.12.1, torchvision 0.27.1, segmentation_models_pytorch 0.5.0, GeoPandas 1.1.4), the exact statistical test used

(scipy.stats.friedmanchisquare), the significance threshold (α = 0.05), and confirmation that no data were missing or excluded from any fold.

---

12. Benjamini–Hochberg controls the FDR, not the FWER

Benjamini–Hochberg controls the false discovery rate, not the family-wise error rate. Revise the terminology. The text should also consistently report adjusted p-values as p_adj.

Corrected on both counts. The text now states that the procedure controls the false discovery rate, and adjusted p-values are reported as p_adj throughout.

---

13. Definition of the effect size *d*

Define the reported effect size d, including the denominator and whether it is paired. Do not label |d|>2 as "statistically significant." Statistical significance and practical effect magnitude should be represented separately.

Both points addressed. The Statistical Analysis subsection now gives an explicit definition: *d* is paired Cohen's d_z, computed as the mean of the per-fold paired differences in IoU relative to the reference configuration, divided by their standard deviation (with Bessel's correction). We verified this definition line-by-line against the analysis script that produced the reported values. We also explain why several effect sizes exceed conventional thresholds despite modest raw differences: with only five paired observations, a small standard deviation of the paired differences can yield a large standardised effect even when the underlying IoU gap is a few percentage points.

The Figure 4 caption previously conflated significance and effect magnitude by marking "Friedman p<0.05 **or** |d|>2" as "statistically significant." This has been rewritten: black borders now indicate statistical significance only (Friedman p_adj < 0.05), and effect sizes are reported separately, with an explicit statement that the two are distinct properties.

---

14. Epoch budget and patience were changed together

The improvement is attributed to relaxed early-stopping patience, but both the epoch budget and patience were changed. The contribution of either change cannot be isolated without an additional controlled comparison.

The reviewer is correct: both parameters were changed simultaneously (from 75 epochs / patience 20 in the ablation to 150 epochs / patience 20 in the operational configuration), and we cannot attribute the improvement to either one individually. The

attribution has been removed and replaced by a neutral statement of what was changed, together with an acknowledgement that isolating the two would require a controlled comparison we did not perform.

---

15. Speculative interpretation of per-fold recall

Interpretations of why individual folds have higher or lower recall are speculative. Either provide evidence based on fold composition or remove these explanations.

Removed. We did not have the fold-composition evidence needed to support those explanations, so we removed them rather than construct a post-hoc justification. The passage now reports the inter-fold variability factually and states explicitly that we do not attempt to attribute it to specific spatial or spectral characteristics.

---

16. Prediction–label mismatch interpreted as newly colonised areas

A mismatch between predictions and temporally inconsistent labels cannot be interpreted as demonstrating detection of newly colonised areas. It may represent expansion, commission error, registration error or label error. The sentence should be revised accordingly.

Agreed, and rewritten. The claim that the model "demonstrates its capacity for targeted generalisation" by detecting newly colonised areas has been removed. The passage now lists the four alternative explanations the reviewer identifies, without arbitrating between them, and states that disentangling them would require contemporaneous field validation that was not available for all sites.

---

17. Duplicate references

References 4 and 34: These appear to be duplicate entries for McLeod et al. Remove one. References 58 and 60: These are duplicate entries for Yeung et al.

Both duplicates removed and the corresponding citations updated to point to the retained entries. While verifying this by recompiling the bibliography, we found two further duplicate entries that had not been flagged (Nie et al. 2023, and Safonova et al. 2023). We removed those as well. Four duplicates have therefore been removed in total.

Editorial requirements

Consent and ethics (requirement 2). This study involved no human subjects, so no informed consent procedure was required. A new "Ethics statement and permits"

subsection in the Methods states this explicitly, and specifies that the land accessed is public (part of the French domaine public maritime), not privately owned.

Permits (requirement 3). Fieldwork consisted of non-invasive vegetation surveys conducted on foot on the intertidal foreshore. Pedestrian access for this type of survey is free under French law and requires no specific authorisation (Article L.321-9 of the Code de l'environnement; Article L.2124-4 of the Code général de la propriété des personnes publiques). Part of the study area overlaps the Natura 2000 site "Rade de Brest, estuaire de l'Aulne" (ZSC FR5300046); the surveys involved no sampling or removal of protected species and did not correspond to listed activities requiring an impact assessment. Drone flights were conducted with the required authorisations from the relevant military and civil aviation authorities. All of this is stated in the new subsection.

Code sharing (requirement 4). The general SemanticSeg4EO experimental framework used to run all experiments in this study remains hosted and version-controlled on GitHub (https://github.com/aleguillou1/SemanticSeg4EO). The scripts specific to the two new experiments presented in this revision (the channel ablation and the leave-one-site-out validation, including the patch-to-site spatial assignment script), together with their outputs, have been added to the project's Zenodo archive (https://zenodo.org/records/18836432).

Preprint and prior submission (requirement 5). Addressed in the cover letter.

Figure copyright (requirements 6 and 7). Figures 1, 2, 8 and 9 use IGN BD ORTHO® aerial imagery, which since 1 January 2021 is open data under the Licence Ouverte / Etalab 2.0. This licence permits free reuse, redistribution and republication including for commercial purposes, subject to attribution, and Etalab states that it is compatible with the Creative Commons Attribution (CC BY) licence family. We have added an explicit attribution line to each affected figure caption and a clarifying statement in the Data Availability section. No Google Maps, Google Earth, or other proprietary imagery is used anywhere in the manuscript. Figure 3 has been entirely redrawn by the authors so that all of its components are original work.

Illegible files (requirement 8). All submission files have been renamed to follow PLOS's required conventions (Manuscript.tex, Manuscript.bib, Fig1.tif–Fig9.tif), which we believe resolves the earlier upload issue.

Additional improvements made during this revision

While preparing this revision we also carried out a full check of the manuscript against PLOS ONE's current formatting requirements, independently of the reviewer's comments, and corrected several issues we found: the abstract exceeded the 300-word limit and has been shortened to 296 words; a footnote (not permitted by PLOS) has been removed; the manuscript now uses double line spacing throughout; a short title has

been added; and heading levels have been reduced from four to the maximum of three permitted.

We hope these revisions address the reviewer's concerns satisfactorily. The two additional experiments in particular have improved the manuscript: they replaced two arguments-by-assertion with measurements, and in both cases the measurements were less flattering than our original framing implied, which we believe makes the paper more useful to readers who might deploy this approach operationally.

Yours sincerely,

Adrien Le Guillou, on behalf of all authors
