# Supplementary material for: Training strategy over architecture: A systematic ablation study for Spartina detection from aerial imagery with small geospatial datasets
Source: PLoS One. 2026 Sep 18;21(9):e0358464. doi: 10.1371/journal.pone.0358464 (PMC13588420; doi:10.1371/journal.pone.0358464)
Supplement: Peer review history

# Peer review: Decision Letter 0

**DOI:** 10.1371/journal.pone.0358464.r001

**Authors:** Judi Hewitt Hewitt

28 Jul 2026

Dear Dr. Le Guillou,

Thank you for submitting your manuscript to PLOS ONE. After careful consideration, we feel that it has merit but does not fully meet PLOS ONE’s publication criteria as it currently stands. Therefore, we invite you to submit a revised version of the manuscript that addresses the points raised during the review process.

Note I was only able to find one reviewer and as they recommended major revisions I have gonr with that rather than hold your publication up any longer.  You may choose to submit elsewhere or back to PlosOne.  If you do send us a revised manuscript please address the reviewer's comments.  If you feel unable to do so or feel that many of them are inappropriate please include some suggested reviewers in your reply.

Please submit your revised manuscript by Sep 25 2026 11:59PM. If you will need more time than this to complete your revisions, please reply to this message or contact the journal office at plosone@plos.org. When you're ready to submit your revision, log on to https://www.editorialmanager.com/pone/ and select the 'Submissions Needing Revision' folder to locate your manuscript file.

- A letter that responds to each point raised by the academic editor and reviewer(s). You should upload this letter as a separate file labeled 'Response to Reviewers'.
- A marked-up copy of your manuscript that highlights changes made to the original version. You should upload this as a separate file labeled 'Revised Manuscript with Track Changes'.
- An unmarked version of your revised paper without tracked changes. You should upload this as a separate file labeled 'Manuscript'.

If you would like to make changes to your financial disclosure, please include your updated statement in your cover letter. Guidelines for resubmitting your figure files are available below the reviewer comments at the end of this letter.

If applicable, we recommend that you deposit your laboratory protocols in protocols.io to enhance the reproducibility of your results. Protocols.io assigns your protocol its own identifier (DOI) so that it can be cited independently in the future. For instructions see: https://journals.plos.org/plosone/s/submission-guidelines#loc-laboratory-protocols. Additionally, PLOS ONE offers an option for publishing peer-reviewed Lab Protocol articles, which describe protocols hosted on protocols.io. Read more information on sharing protocols at https://plos.org/protocols?utm_medium=editorial-email&utm_source=authorletters&utm_campaign=protocols.

As the corresponding author, your ORCID iD is verified in the submission system and will appear in the published article. PLOS supports the use of ORCID, and we encourage all coauthors to register for an ORCID iD and use it as well. Please encourage your coauthors to verify their ORCID iD within the submission system before final acceptance, as unverified ORCID iDs will not appear in the published article. *Only*  the individual author can complete the verification step; PLOS staff *cannot*  verify ORCID iDs on behalf of authors.

We look forward to receiving your revised manuscript.

Kind regards,

Judi Hewitt, Hewitt

Academic Editor

PLOS One

Journal Requirements:

When submitting your revision, we need you to address these additional requirements.

1. Please ensure that your manuscript meets PLOS ONE's style requirements, including those for file naming. The PLOS ONE style templates can be found at

https://journals.plos.org/plosone/s/file?id=wjVg/PLOSOne_formatting_sample_main_body.pdf and https://journals.plos.org/plosone/s/file?id=ba62/PLOSOne_formatting_sample_title_authors_affiliations.pdf

2. Please provide additional details regarding participant consent. In the ethics statement in the Methods and online submission information, please ensure that you have specified (1) whether consent was informed and (2) what type you obtained (for instance, written or verbal, and if verbal, how it was documented and witnessed). If your study included minors, state whether you obtained consent from parents or guardians. If the need for consent was waived by the ethics committee, please include this information.

If you are reporting a retrospective study of medical records or archived samples, please ensure that you have discussed whether all data were fully anonymized before you accessed them and/or whether the IRB or ethics committee waived the requirement for informed consent. If patients provided informed written consent to have data from their medical records used in research, please include this information.

3. In your Methods section, please provide additional information regarding the permits you obtained for the work. Please ensure you have included the full name of the authority that approved the field site access and, if no permits were required, a brief statement explaining why.

4. Please note that PLOS One has specific guidelines on code sharing for submissions in which author-generated code underpins the findings in the manuscript. In these cases, we expect all author-generated code to be made available without restrictions upon publication of the work. Please review our guidelines at https://journals.plos.org/plosone/s/materials-and-software-sharing#loc-sharing-code and ensure that your code is shared in a way that follows best practice and facilitates reproducibility and reuse.

5. We noted in your submission details that a portion of your manuscript may have been presented or published elsewhere. A preprint of a previous version of this manuscript is publicly available on SSRN (https://papers.ssrn.com/sol3/papers.cfm?abstract_id=6346443), and is also indexed on HAL and ResearchGate. The current submission has been substantially revised relative to that preprint, notably in the framing of the operational scope, the spatial autocorrelation analysis, and the discussion of generalisability. Consistent with PLOS policy on preprints, this prior posting is not considered prior publication. An earlier version of this work was submitted to ISPRS Journal of Photogrammetry and Remote Sensing and rejected following peer review; the manuscript was subsequently comprehensively revised before the current submission.

Please clarify whether this [conference proceeding or publication] was peer-reviewed and formally published. If this work was previously peer-reviewed and published, in the cover letter please provide the reason that this work does not constitute dual publication and should be included in the current manuscript.

6. We note that Figure(s) 1, 2, 8, 9, in your submission contain [map/satellite] images which may be copyrighted. All PLOS content is published under the Creative Commons Attribution License (CC BY 4.0), which means that the manuscript, images, and Supporting Information files will be freely available online, and any third party is permitted to access, download, copy, distribute, and use these materials in any way, even commercially, with proper attribution. For these reasons, we cannot publish previously copyrighted maps or satellite images created using proprietary data, such as Google software (Google Maps, Street View, and Earth). For more information, see our copyright guidelines: http://journals.plos.org/plosone/s/licenses-and-copyright.

We require you to either (1) present written permission from the copyright holder to publish these figures specifically under the CC BY 4.0 license, or (2) remove the figures from your submission:

a. You may seek permission from the original copyright holder of Figure(s) 1, 2, 8, 9, to publish the content specifically under the CC BY 4.0 license.

We recommend that you contact the original copyright holder with the Content Permission Form (http://journals.plos.org/plosone/s/file?id=7c09/content-permission-form.pdf) and the following text:

“I request permission for the open-access journal PLOS ONE to publish XXX under the Creative Commons Attribution License (CCAL) CC BY 4.0 (http://creativecommons.org/licenses/by/4.0/). Please be aware that this license allows unrestricted use and distribution, even commercially, by third parties. Please reply and provide explicit written permission to publish XXX under a CC BY license and complete the attached form.”

Please upload the completed Content Permission Form or other proof of granted permissions as an "Other" file with your submission.

In the figure caption of the copyrighted figure, please include the following text: “Reprinted from [ref] under a CC BY license, with permission from [name of publisher], original copyright [original copyright year].”

b. If you are unable to obtain permission from the original copyright holder to publish these figures under the CC BY 4.0 license or if the copyright holder’s requirements are incompatible with the CC BY 4.0 license, please either i) remove the figure or ii) supply a replacement figure that complies with the CC BY 4.0 license. Please check copyright information on all replacement figures and update the figure caption with source information. If applicable, please specify in the figure caption text when a figure is similar but not identical to the original image and is therefore for illustrative purposes only.

The following resources for replacing copyrighted map figures may be helpful:

USGS National Map Viewer (public domain): http://viewer.nationalmap.gov/viewer/

The Gateway to Astronaut Photography of Earth (public domain): http://eol.jsc.nasa.gov/sseop/clickmap/

Maps at the CIA (public domain): https://www.cia.gov/library/publications/the-world-factbook/index.html and https://www.cia.gov/library/publications/cia-maps-publications/index.html

NASA Earth Observatory (public domain): http://earthobservatory.nasa.gov/

Landsat: http://landsat.visibleearth.nasa.gov/

USGS EROS (Earth Resources Observatory and Science (EROS) Center) (public domain): http://eros.usgs.gov/#

Natural Earth (public domain): http://www.naturalearthdata.com/

7. We note that Figure 3 in your submission contain copyrighted images. All PLOS content is published under the Creative Commons Attribution License (CC BY 4.0), which means that the manuscript, images, and Supporting Information files will be freely available online, and any third party is permitted to access, download, copy, distribute, and use these materials in any way, even commercially, with proper attribution. For more information, see our copyright guidelines: http://journals.plos.org/plosone/s/licenses-and-copyright.

We require you to either (1) present written permission from the copyright holder to publish these figures specifically under the CC BY 4.0 license, or (2) remove the figures from your submission:

a. You may seek permission from the original copyright holder of Figure 3 to publish the content specifically under the CC BY 4.0 license.

We recommend that you contact the original copyright holder with the Content Permission Form (http://journals.plos.org/plosone/s/file?id=7c09/content-permission-form.pdf) and the following text:

“I request permission for the open-access journal PLOS ONE to publish XXX under the Creative Commons Attribution License (CCAL) CC BY 4.0 (http://creativecommons.org/licenses/by/4.0/). Please be aware that this license allows unrestricted use and distribution, even commercially, by third parties. Please reply and provide explicit written permission to publish XXX under a CC BY license and complete the attached form.”

Please upload the completed Content Permission Form or other proof of granted permissions as an "Other" file with your submission.

In the figure caption of the copyrighted figure, please include the following text: “Reprinted from [ref] under a CC BY license, with permission from [name of publisher], original copyright [original copyright year].”

b. If you are unable to obtain permission from the original copyright holder to publish these figures under the CC BY 4.0 license or if the copyright holder’s requirements are incompatible with the CC BY 4.0 license, please either i) remove the figure or ii) supply a replacement figure that complies with the CC BY 4.0 license. Please check copyright information on all replacement figures and update the figure caption with source information. If applicable, please specify in the figure caption text when a figure is similar but not identical to the original image and is therefore for illustrative purposes only.

8. We are unable to open your Supporting Information file [article_plos_one_final (1).tex, plos_bibtex (1).bib]. Please kindly revise as necessary and re-upload.

9. If the reviewer comments include a recommendation to cite specific previously published works, please review and evaluate these publications to determine whether they are relevant and should be cited. There is no requirement to cite these works unless the editor has indicated otherwise.

[Note: HTML markup is below. Please do not edit.]

Reviewers' comments:

Reviewer's Responses to Questions

**Comments to the Author**

1. Is the manuscript technically sound, and do the data support the conclusions?

Reviewer #1: Partly

**********

2. Has the statistical analysis been performed appropriately and rigorously?

Reviewer #1: Yes

**********

3. Have the authors made all data underlying the findings in their manuscript fully available?

The PLOS Data policy

Reviewer #1: Yes

**********

4. Is the manuscript presented in an intelligible fashion and written in standard English?

Reviewer #1: Yes

**********

Reviewer #1: The manuscript addresses an important and practical problem: automated mapping of Spartina/Sporobolus in small, spatially fragmented intertidal systems using routinely available aerial imagery and LiDAR-derived elevation data. The ablation design is ambitious and useful: three segmentation architectures, multiple training-strategy axes, five-fold cross-validation, and operational wall-to-wall inference. There are some points that the authors should take into account which can potentially further improve the paper.

Major concerns.

Lines 186–203; The input data description was not clear to me. The manuscript uses R, G, NIR, and DSM inputs, but does not demonstrate the individual contribution of spectral information and elevation information. A key missing experiment is comparison among CIR-only, DSM-only, CIR+DSM, and potentially classical machine-learning baselines. Without this, it remains unclear whether the observed performance is due to deep learning architecture or simply the strong ecological signal provided by elevation.

Line 218 – 248. I am a bit concerned about the spatial cross-validation analysis. The current validation strategy may still overestimate model transferability because training and validation patches are spatially close and originate from the same sites. Although the manuscript evaluates spatial autocorrelation, neighbouring patches remain available during training and validation. Please consider adding leave-one-site-out validation, spatial block cross-validation, or distance-buffered validation to better assess performance under realistic deployment conditions.

Minor concerns

Lines 1–2 and throughout: Taxonomic terminology is inconsistent. The manuscript alternates among Spartina, Spartina alterniflora, Sporobolus and Sporobolus alterniflorus. Introduce the accepted name and synonym once, and then use one name consistently.

Lines 17–18: “Obione” is used while later sections use Halimione portulacoides. Use consistent taxonomic terminology. Also reconsider describing all the listed plants or communities as “endemic.”

Lines 117–127: The study-site description lists Daoulas, Mengleuz/Rosméléc and Le Faou, whereas Figure 2 and the dataset section also include Le Pédel, Pont-Callec and Hôpital-Camfrout. Describe all training, validation and test sites consistently.

Lines 140–160: The text states that labels were assembled from “three complementary sources,” but these sources and their temporal coverage are not clearly enumerated. A table should list each site, imagery date, field/drone date, labelling source and reference quality.

Line 158: A digitisation scale of 1:144. Please verify the value and explain how digitisation consistency was assessed.

Lines 164–198: The input is first called a “normalised digital surface model,” but it is subsequently described as absolute surface elevation. An nDSM and a DSM are different products. Use the correct term throughout.

Lines 173–180: The claim that a model trained on 20-cm aerial CIR imagery can be directly extrapolated to Pleiades, SPOT-7 or Sentinel-2 is too strong. These sensors differ substantially in spatial resolution, spectral response and acquisition geometry. Present this as a potential future transfer experiment requiring retraining and validation.

Lines 186–201: State the LiDAR acquisition dates. Resampling a 50-cm DSM to 20 cm does not produce an effective spatial resolution of 20 cm, so this distinction should be made explicit.

Lines 205–209 versus 224–227: The dataset is described as containing approximately 30% Spartina pixels, whereas the fold analysis reports approximately 24.8%. Explain whether different spatial masks, subsets or denominators were used.

Methods section: Essential training details are missing, including maximum epochs, early-stopping patience etc. Early stopping and a maximum of 375 cumulative epochs appear only later in the Results.

Lines 333–337: Benjamini–Hochberg controls the false discovery rate, not the family-wise error rate. Revise the terminology. The text should also consistently report adjusted p-values as p_adj.

Figure 4: Define the reported effect size d, including the denominator and whether it is paired. Do not label ∣d∣>2as “statistically significant.” Statistical significance and practical effect magnitude should be represented separately.

Lines 437–444: The improvement is attributed to relaxed early-stopping patience, but both the epoch budget and patience were changed. The contribution of either change cannot be isolated without an additional controlled comparison.

Lines 455–458: Interpretations of why individual folds have higher or lower recall are speculative. Either provide evidence based on fold composition or remove these explanations.

Lines 510–517: A mismatch between predictions and temporally inconsistent labels cannot be interpreted as demonstrating detection of newly colonised areas. It may represent expansion, commission error, registration error or label error. The sentence should be revised accordingly.

References 4 and 34: These appear to be duplicate entries for McLeod et al. Remove one.

References 58 and 60: These are duplicate entries for Yeung et al.

**********

what does this mean?). If published, this will include your full peer review and any attached files.

If you choose “no”, your identity will remain anonymous but your review may still be made public.

**Do you want your identity to be public for this peer review?**  For information about this choice, including consent withdrawal, please see our Privacy Policy

Reviewer #1: No

**********

[NOTE: If reviewer comments were submitted as an attachment file, they will be attached to this email and accessible via the submission site. Please log into your account, locate the manuscript record, and check for the action link "View Attachments". If this link does not appear, there are no attachment files.]

To ensure your figures meet our technical requirements, please review our figure guidelines: https://journals.plos.org/plosone/s/figures

You may also use PLOS’s free figure tool, NAAS, to help you prepare publication quality figures: https://journals.plos.org/plosone/s/figures#loc-tools-for-figure-preparation.

NAAS will assess whether your figures meet our technical requirements by comparing each figure against our figure specifications.

# Author comment: Author response to Decision Letter 1

**DOI:** 10.1371/journal.pone.0358464.r002

20 Aug 2026

RESPONSE TO REVIEWERS

Manuscript: PONE-D-26-28775

Title: Training strategy over architecture: A systematic ablation study for Spartina detection from aerial imagery with small geospatial datasets

----------------------------------------------------------------------

Dear Dr. Hewitt, dear Reviewer,

Thank you for the careful reading of our manuscript and for the constructive comments. We are particularly grateful to Reviewer 1, whose two major points led us to run additional experiments that materially changed how we interpret and present our results. In both cases the new experiments confirmed the reviewer's concern rather than dismissing it, and we have revised the manuscript accordingly, including the abstract.

Below we respond to each point in turn. Reviewer comments are given in italics, our responses in plain text, with the corresponding manuscript changes indicated. Table and figure numbers refer to the final revised manuscript.

----------------------------------------------------------------------

REVIEWER 1 — MAJOR CONCERNS

===========================

1. INDIVIDUAL CONTRIBUTION OF SPECTRAL AND ELEVATION INFORMATION

The manuscript uses R, G, NIR, and DSM inputs, but does not demonstrate the individual contribution of spectral information and elevation information. A key missing experiment is comparison among CIR-only, DSM-only, CIR+DSM, and potentially classical machine-learning baselines. Without this, it remains unclear whether the observed performance is due to deep learning architecture or simply the strong ecological signal provided by elevation.

We agree that this experiment was missing, and we have now run it. We retrained the selected operational configuration under three input regimes that differ only in the channels supplied to the network, holding everything else constant (same architecture, same training strategy, same five-fold partition, channel selection applied before per-patch normalisation to avoid cross-contamination between regimes).

Input IoU (%) F1 (%)

CIR + DSM (reference) 76.7 +/- 2.1 86.8 +/- 1.3

CIR-only (R, G, NIR) 75.0 +/- 2.8 85.7 +/- 1.8

DSM-only (elevation) 54.2 +/- 3.1 70.2 +/- 2.6

The answer to the reviewer's question is that the reported performance is driven predominantly by the spectral channels, not by elevation: removing the DSM entirely costs only 1.7 pts IoU. This is a correction to the framing of our original manuscript, which presented surface elevation as "a strong discriminant"; that claim is not supported by the data and has been rewritten as an expectation to be tested rather than an established fact.

We were careful not to over-correct in the other direction. Elevation alone reaches 54.2% IoU, more than twice what a degenerate all-positive predictor would obtain at our class prevalence (24.8%), so the DSM does carry real discriminative signal; its precision is also the lowest and most variable of the three regimes, indicating it produces meaningfully more commission errors when used alone. We discuss this apparent tension explicitly in a new Discussion subsection, and propose that the small marginal contribution of the DSM in the combined configuration reflects, at least in part, the limitations of input-level (early) fusion of a physically heterogeneous modality into an encoder pre-trained on three-channel imagery: the modified input layer cannot fully inherit the pre-trained weights, and the DSM channel must be initialised by an arbitrary heuristic (replicating a spectral channel's weights) that fine-tuning must subsequently undo, whereas the CIR-only model inherits its weights unmodified. We support this reading with three published sources on multi-modal fusion in segmentation (El Madawi et al. 2019; Xiong et al. 2023; Hurtado & Valada 2022), and state explicitly that we cannot separate genuine spectral–elevation redundancy from fusion inefficiency with the experiments reported here, identifying improved fusion strategies as a priority for future work.

Regarding classical machine-learning baselines: we read the reviewer's "potentially" as marking this as optional, and have not added them, in order to keep the revision focused on the two experiments that directly address the stated concerns. We would be willing to add them if the reviewer considers them necessary.

Changes: New Results subsection "Contribution of spectral and elevation inputs (channel ablation)" with Table 9; new Discussion subsection "Interpreting the modest marginal contribution of elevation: an input-level fusion limitation"; the Methods description of the DSM rewritten to remove the unsupported "strong discriminant" claim; three new references added; the abstract now reports the channel ablation.

----------------------------------------------------------------------

2. SPATIAL CROSS-VALIDATION AND TRANSFERABILITY

I am a bit concerned about the spatial cross-validation analysis. The current validation strategy may still overestimate model transferability because training and validation patches are spatially close and originate from the same sites. [...] Please consider adding leave-one-site-out validation, spatial block cross-validation, or distance-buffered validation to better assess performance under realistic deployment conditions.

The reviewer is right, and our original response to this concern (a set of qualitative mitigating arguments explaining why leave-one-site-out had not been adopted) was not an adequate substitute for the experiment itself. We have now run the leave-one-site-out (LOSO) validation.

Each of the labelled sites was withheld in its entirety as validation, with the model retrained from scratch on the remaining sites. Because our patch filenames are purely sequential and carry no site information, site membership was determined from the georeferenced extent of each patch via a spatial join against the site boundary polygons; all 810 patches were successfully assigned.

The result confirms the concern:

- Mean IoU drops from 76.7 ± 2.1% (random five-fold) to 64.2 ± 9.3% (LOSO), a −12.5 pts shift, with the standard deviation across folds increasing roughly 4.4-fold.

- Per-site performance ranges from 50.5% IoU (Pont-Callec) to 79.5% IoU (Le Pédel), a 28.9-point spread that random cross-validation cannot expose by construction.

- The variability is concentrated in the Spartina class; the background class remains comparatively stable (84.6 ± 5.1%).

We now present the random five-fold estimates as an optimistic, same-distribution estimate appropriate for sites resembling those already mapped, and the LOSO estimates as the stricter estimate to be preferred whenever site-level extrapolation is intended. The abstract, the operational implications paragraph, and the Discussion have all been revised accordingly; in particular, the sentence stating that the predicted surfaces "can be trusted as reliable estimates" has been qualified to specify sites resembling those already mapped.

We also report honestly what we could not explain. Pont-Callec generalises poorly, but we found no factor fully consistent with the pattern: Pont-Callec and Le Pédel share the same ground-truth labelling source (photo-interpretation only, no drone) yet sit at opposite extremes of the LOSO range. We report this as an open empirical finding rather than attribute it to a mechanism we cannot demonstrate.

One limitation is stated explicitly: the LOSO experiment was run on the selected operational configuration only, not repeated across the full ablation matrix. Our argument that the relative ranking of training strategies is robust to autocorrelation bias is therefore not directly tested by these results, and we now say so rather than leaving it implicit.

Changes: New Results subsection "Leave-one-site-out validation of the operational configuration" with Table 10; Discussion subsection on spatial autocorrelation substantially rewritten (the previous justification for not performing LOSO has been replaced by the results and their interpretation); abstract and operational implications revised.

----------------------------------------------------------------------

REVIEWER 1 — MINOR CONCERNS

===========================

3. TAXONOMIC TERMINOLOGY

The manuscript alternates among Spartina, Spartina alterniflora, Sporobolus and Sporobolus alterniflorus. Introduce the accepted name and synonym once, and then use one name consistently.

Corrected. The accepted name and its synonym are introduced once, at first mention in the Introduction (Sporobolus alterniflorus, syn. Spartina alterniflora), and "Spartina" is used consistently everywhere else, including the abstract, figure captions, and conclusion.

----------------------------------------------------------------------

4. "OBIONE" VS HALIMIONE PORTULACOIDES; "ENDEMIC"

"Obione" is used while later sections use Halimione portulacoides. Use consistent taxonomic terminology. Also reconsider describing all the listed plants or communities as "endemic."

Both corrected. "Obione" has been replaced by Halimione portulacoides throughout. The "endemic" qualifier has been removed from the list of co-occurring halophytes, where it was not justified; it is retained only for Limonium humile, where it is accurate.

----------------------------------------------------------------------

5. INCONSISTENT LIST OF STUDY SITES

The study-site description lists Daoulas, Mengleuz/Rosméléc and Le Faou, whereas Figure 2 and the dataset section also include Le Pédel, Pont-Callec and Hôpital-Camfrout. Describe all training, validation and test sites consistently.

This was a real inconsistency and we have rewritten the study-site section to resolve it. All six labelled zones are now listed and described explicitly: Daoulas, Mengleuz/Rosmélec, Le Faou, Keroullé, Le Pédel, and Pont-Callec. Hôpital-Camfrout is described separately as the independent test site, since it contributes no labelled training patches.

We also identified and documented a source of ambiguity that had contributed to the confusion, including in our own reporting: Mengleuz/Rosmélec is a distinct inlet but lies within the broader Daoulas estuarine system, and is therefore sometimes reported as part of Daoulas. This is now stated explicitly at the start of the study-site section.

While preparing the leave-one-site-out analysis we further found that the "Le Faou" column of the fold composition table (Table 2) aggregated Le Faou (283 patches) and the adjacent Keroullé inlet (58 patches). We verified this arithmetically against the georeferenced site assignment: all other columns match exactly under both assignments. Rather than silently altering the table, we documented this aggregation in a table footnote and report the two zones separately in the LOSO analysis (Table 10), where site membership was reassigned from the georeferenced extent of each patch.

----------------------------------------------------------------------

6. DIGITISATION SCALE OF 1:144

A digitisation scale of 1:144. Please verify the value and explain how digitisation consistency was assessed.

We have verified the value: 1:144 is correct and intentional. It was adopted to match the scale used by Le Meur (2024) for the same estuarine sites, so that the resulting layers remain directly comparable with that earlier mapping. We have added an explanation of how consistency was ensured: digitisation was performed at this fixed reference scale rather than at a variable, screen-zoom-dependent scale, following the same protocol as the earlier study.

----------------------------------------------------------------------

7. NDSM VS DSM

The input is first called a "normalised digital surface model," but it is subsequently described as absolute surface elevation. An nDSM and a DSM are different products. Use the correct term throughout.

Corrected. The product used is an absolute DSM, not a normalised nDSM. The residual occurrence of "normalised" has been removed; the term "DSM" is now used consistently.

----------------------------------------------------------------------

8. EXTRAPOLATION TO PLÉIADES, SPOT-7, SENTINEL-2

The claim that a model trained on 20-cm aerial CIR imagery can be directly extrapolated to Pleiades, SPOT-7 or Sentinel-2 is too strong. [...] Present this as a potential future transfer experiment requiring retraining and validation.

Agreed and rewritten. The passage no longer claims direct extrapolation. It now states that these sensors differ substantially in spatial resolution, spectral response, and acquisition geometry, that transfer would require dedicated retraining and independent validation on sensor-specific data, and that we identify this as a direction for future work rather than a capability demonstrated here.

----------------------------------------------------------------------

9. LIDAR ACQUISITION DATES AND EFFECTIVE RESOLUTION

State the LiDAR acquisition dates. Resampling a 50-cm DSM to 20 cm does not produce an effective spatial resolution of 20 cm, so this distinction should be made explicit.

The acquisition campaign has been added: IGN LiDAR HD national programme, Brittany acquisition campaign, winter 2024–2025. We note that IGN publishes day-level acquisition dates only per individual 1 km tile, not per region; we report the campaign period, which is the level at which the information is authoritatively available for the study area.

On the resampling point, the reviewer is correct and the manuscript already distinguished the native 50 cm resolution from the 20 cm resampled grid; we have kept this distinction explicit, stating that bilinear interpolation to 20 cm GSD was performed to match the BD ORTHO® grid for stacking purposes and does not create additional elevation detail.

----------------------------------------------------------------------

10. CLASS IMBALANCE: 30% VS 24.8%

The dataset is described as containing approximately 30% Spartina pixels, whereas the fold analysis reports approximately 24.8%. Explain whether different spatial masks, subsets or denominators were used.

There was no methodological difference; the "≈30%" figure was an imprecise approximation carried over from an earlier draft. The measured value is 24.8%, as reported in the fold composition table. We have harmonised all occurrences in the manuscript to 24.8%.

----------------------------------------------------------------------

11. MISSING TRAINING DETAILS IN METHODS

Essential training details are missing, including maximum epochs, early-stopping patience etc. Early stopping and a maximum of 375 cumulative epochs appear only later in the Results.

Added. The Methods now state, for the ablation study, a maximum of 75 training epochs with an early-stopping patience of 20 epochs (monitored on validation IoU), both in the text and in the Table 3 caption. We also clarified that the "375 epochs" figure reported in the Results is a cumulative total across the five folds (5 × 75 maximum), not a per-fold maximum, which was ambiguous in the original text.

We have additionally added a dedicated "Statistical analysis" subsection to the Methods, reporting the exact software used (Python 3.12.13, NumPy 2.4.4, pandas 3.0.3, SciPy 1.18.0, scikit-learn 1.9.0, PyTorch 2.12.1, torchvision 0.27.1, segmentation_models_pytorch 0.5.0, GeoPandas 1.1.4), the exact statistical test used (`scipy.stats.friedmanchisquare`), the significance threshold (α = 0.05), and confirmation that no data were missing or excluded from any fold.

----------------------------------------------------------------------

12. BENJAMINI–HOCHBERG CONTROLS THE FDR, NOT THE FWER

Benjamini–Hochberg controls the false discovery rate, not the family-wise error rate. Revise the terminology. The text should also consistently report adjusted p-values as p_adj.

Corrected on both counts. The text now states that the procedure controls the false discovery rate, and adjusted p-values are reported as p_adj throughout.

----------------------------------------------------------------------

13. DEFINITION OF THE EFFECT SIZE D

Define the reported effect size d, including the denominator and whether it is paire

Attachment Submitted filename: Response_to_Reviewers.pdf

# Peer review: Decision Letter 1

**DOI:** 10.1371/journal.pone.0358464.r003

**Authors:** Judi Hewitt Hewitt

2 Sep 2026

Training strategy over architecture: A systematic ablation study for Spartina detection from aerial imagery with small geospatial datasets

PONE-D-26-28775R1

Dear Dr. Le Guillou,

We’re pleased to inform you that your manuscript has been judged scientifically suitable for publication and will be formally accepted for publication once it meets all outstanding technical requirements.

Within one week, you’ll receive an e-mail detailing the required amendments. When these have been addressed, you’ll receive a formal acceptance letter and your manuscript will be scheduled for publication.

An invoice will be generated when your article is formally accepted. Please note, if your institution has a publishing partnership with PLOS and your article meets the relevant criteria, all or part of your publication costs will be covered. Please make sure your user information is up-to-date by logging into Editorial Manager at Editorial Manager® and clicking the ‘Update My Information' link at the top of the page. For questions related to billing, please contact billing support.

If your institution or institutions have a press office, please notify them about your upcoming paper to help maximize its impact. If they’ll be preparing press materials, please inform our press team as soon as possible -- no later than 48 hours after receiving the formal acceptance. Your manuscript will remain under strict press embargo until 2 pm Eastern Time on the date of publication. For more information, please contact onepress@plos.org.

Kind regards,

Judi Hewitt, Hewitt

Academic Editor

PLOS One

Additional Editor Comments (optional):

Reviewers' comments:

Reviewer's Responses to Questions

**Comments to the Author**

Reviewer #1: All comments have been addressed

**********

2. Is the manuscript technically sound, and do the data support the conclusions?

Reviewer #1: Yes

**********

3. Has the statistical analysis been performed appropriately and rigorously?

Reviewer #1: Yes

**********

4. Have the authors made all data underlying the findings in their manuscript fully available?

The PLOS Data policy

Reviewer #1: Yes

**********

5. Is the manuscript presented in an intelligible fashion and written in standard English?

Reviewer #1: Yes

**********

Reviewer #1: (No Response)

**********

what does this mean?). If published, this will include your full peer review and any attached files.

If you choose “no”, your identity will remain anonymous but your review may still be made public.

**Do you want your identity to be public for this peer review?**  For information about this choice, including consent withdrawal, please see our Privacy Policy

Reviewer #1: No

**********

# Editor report: Acceptance letter

**DOI:** 10.1371/journal.pone.0358464.r004

**Authors:** Judi Hewitt Hewitt

PONE-D-26-28775R1

PLOS One

Dear Dr. Le Guillou,

I'm pleased to inform you that your manuscript has been deemed suitable for publication in PLOS One. Congratulations! Your manuscript is now being handed over to our production team.

At this stage, our production department will prepare your paper for publication. This includes ensuring the following:

* All references, tables, and figures are properly cited

* All relevant supporting information is included in the manuscript submission,

* There are no issues that prevent the paper from being properly typeset

You will receive further instructions from the production team, including instructions on how to review your proof when it is ready. Please keep in mind that we are working through a large volume of accepted articles, so please give us a few days to review your paper and let you know the next and final steps.

Lastly, if your institution or institutions have a press office, please let them know about your upcoming paper now to help maximize its impact. If they'll be preparing press materials, please inform our press team within the next 48 hours. Your manuscript will remain under strict press embargo until 2 pm Eastern Time on the date of publication. For more information, please contact onepress@plos.org.

You will receive an invoice from PLOS for your publication fee after your manuscript has reached the completed accept phase. If you receive an email requesting payment before acceptance or for any other service, this may be a phishing scheme. Learn how to identify phishing emails and protect your accounts at https://explore.plos.org/phishing.

If we can help with anything else, please email us at customercare@plos.org.

Thank you for submitting your work to PLOS One and supporting open access.

Kind regards,

PLOS One Editorial Office Staff

on behalf of

Dr. Judi Hewitt, Hewitt

Academic Editor

PLOS One
